# Coffee (*Coffea arabica* L.) Silverskin: Transforming a Recycled By‐Product Into Natural Antioxidant Ingredients for Ready‐to‐Eat Fish Products

**DOI:** 10.1111/1750-3841.71577

**Published:** 2026-10-21

**Authors:** Antonia Dayane Jenyffer de Farias Marques, Mario Estevez, José María Martín‐Miguélez, Andrea Sánchez‐Canales, Eike Guilherme Torres de Souza, Sonia Ventanas, Josué Delgado, Mercia de Sousa Galvão, Marcos dos Santos Lima, Taliana Kênia Alencar Bezerra, David Morcuende

**Affiliations:** ^1^ Department of Food Engineering Technology Centre, Federal University of Paraiba Joao Pessoa Brazil; ^2^ IPROCAR Research Institute, TECAL Research Group Universidad de Extremadura Cáceres Spain; ^3^ Department of Food Technology Federal Institute of Sertão Pernambucano Petrolina Brazil

**Keywords:** antioxidant, coffee roasting by‐products, oxidative stability

## Abstract

Given salmon's high susceptibility to oxidation and the need for sustainable food processing, this study evaluated the technological potential of incorporating 0.5% coffee silverskin (CSS), using two specific materials evaluated in this study, namely, artisanal (ACS) and industrial (ICS) coffee silverskins, into restructured salmon stored under refrigeration (3°C) for 28 days (sensory analysis was limited to 14 days). ACS showed higher 2,2‐diphenyl‐1‐picrylhydrazyl (DPPH) and ABTS scavenging activity and chlorogenic acid content, whereas ICS exhibited greater ferric reducing antioxidant power (FRAP), consistent with Maillard reaction products. Regardless of antioxidant mechanism, CSS reduced lipid oxidation, lowering malondialdehyde (MDA) and volatile oxidation markers. However, 0.5% CSS did not prevent apparent protein carbonylation, with treated groups showing higher 2,4‐dinitrophenylhydrazine (DNPH)‐reactive carbonyl values during late storage. Although CSS darkened the product, trained‐panel liking was maintained, whereas rancidity and warmed‐over flavor (WOF) were reduced compared to the control, although sensory patterns varied throughout storage. These findings identify CSS as a potential natural antioxidant for enhancing oxidative stability and supporting the sustainable valorization of coffee industry by‐products, although further optimization may be needed to overcome sensory limitations and support its broader application.

**Practical Applications:**

Coffee silverskin (CSS), a coffee roasting by‐product, is a potential natural antioxidant for improving oxidative stability and delaying rancidity in ready‐to‐eat restructured fish. Its comparative performance across the two evaluated sources supports its potential use as a clean‐label antioxidant ingredient while promoting circular economy practices.

## 1 Introduction

Recycling food by‐products is a promising strategy for reducing waste and the environmental impact of food production (Thorsen et al. 2024). According to the Food and Agriculture Organization (FAO) (2024), approximately one‐third of the food produced globally is lost or underutilized, accounting for 8%–10% of global greenhouse gas emissions. Reusing agro‐industrial by‐products reduces loss and enables the development of innovative, functional, and sustainable foods while supporting circular food production systems (Martuscelli et al. 2021).

The production of coffee generates a significant amount of by‐products that are often discarded or underexploited. However, these by‐products represent valuable resources for food applications (Bondam et al. 2022). One such by‐product is coffee silverskin (CSS), a thin layer that covers green coffee beans and detaches during roasting. CSS can be obtained on an industrial scale through increased coffee production efficiency or on a smaller, artisanal scale to cater markets that value natural and sustainable products (Cirne et al. 2019). The global roasting industry is estimated to generate approximately 400,000 t of CSS annually (Nolasco et al. 2022). Crucially, the chemical composition and bioactive potential of coffee by‐products are strongly influenced by roasting conditions, meaning that artisanal and industrial processes yield distinct chemical markers and functional properties (Alcantara et al. 2025).

Recent studies have demonstrated that CSS contains phenolic compounds with antioxidant properties, primarily including chlorogenic acids, which exhibit strong free radical‐scavenging capacities (Nzekoue et al. 2020; Martuscelli et al. 2021). Furthermore, thermal processing during roasting promotes the Maillard reaction, generating non‐phenolic antioxidant compounds such as reductones and high‐molecular‐weight melanoidins (Bolchini et al. 2025). These bioactive constituents may be strategically applied in food systems susceptible to oxidative deterioration, such as fish and fish‐derived products (Cilli et al. 2020). Salmon, for instance, is an excellent source of protein and polyunsaturated fatty acids, including omega‐3 fatty acids, yet it is highly susceptible to lipid oxidation. This instability, combined with processing variables, may compromise its sensory attributes, nutritional value, and storage stability (Mozzon et al. 2024; Haq et al. 2025).

The storage stability of restructured fish products may be challenged by lipid oxidation and associated sensory deterioration, which can compromise product quality during refrigerated storage (Tokay et al. 2021; Gautam et al. 2025). These limitations are relevant because the restructuring process modifies the characteristics of fish muscle, including texture and shape, and may create a matrix in which oxidative and sensory stability need to be carefully controlled (Ren et al. 2024). The incorporation of functional ingredients with antioxidant properties may, therefore, help mitigate oxidative deterioration in these products during storage (Sánchez‐Alonso et al. 2011). Within a circular economy framework, CSS has shown potential as a versatile clean‐label ingredient for the food industry (Mesárošová et al. 2025; Özhamamcı and Sayın 2025; Zoccatelli and Ciulu 2025).

Although previous studies have demonstrated the antioxidant effectiveness of CSS in meat‐based products (Martuscelli et al. 2021; Mesárošová et al. 2025), its application in fish‐based foods has received little attention. This gap is particularly relevant because fish lipids are rich in polyunsaturated fatty acids and therefore highly susceptible to oxidative deterioration. Coffee‐derived phenolic compounds, including chlorogenic and caffeic acids, have been reported to inhibit lipid oxidation in fish muscle models (Medina et al. 2007; Cao et al. 2019), suggesting that phenolic‐rich coffee by‐products may provide similar protection.

However, the antioxidant performance of CSS in fish‐based matrices, particularly restructured products, remains poorly understood. Moreover, roasting conditions are known to influence CSS composition by degrading heat‐sensitive phenolics, particularly chlorogenic acids, while promoting the formation of Maillard reaction products such as melanoidins (Gemechu 2020; Wu et al. 2022). Consequently, the two CSS sources evaluated in this study, artisanal coffee silverskin (ACS) and industrial coffee silverskin (ICS), may exhibit distinct chemical profiles and antioxidant performance. Nevertheless, the implications of these compositional variations for the oxidative and sensory stability of restructured fish products remain unclear.

From this perspective, research focusing on the impact of plant‐derived antioxidants, such as coffee CSS, on oxidation‐prone matrices is of great importance (Estévez 2021; Montenegro Gallazzi et al. 2025). Salmon was selected as the study material because its high content of polyunsaturated fatty acids, particularly omega‐3 fatty acids, makes it highly susceptible to lipid oxidation, providing a suitable matrix for evaluating the antioxidant effects of CSS (Alhammad et al. 2021; R. Ferreira et al. 2022; Mozzon et al. 2024).

The first hypothesis of this study is that CSS can act as a natural antioxidant ingredient capable of delaying oxidative deterioration in restructured salmon. The second hypothesis is that ACS and ICS will exhibit distinct effects on oxidative and sensory quality due to differences in their roasting‐induced chemical profiles. Therefore, the present study evaluated the effects of ACS and ICS on the oxidative and sensory stability of restructured salmon during refrigerated storage.

## 2 Materials and Methods

### 2.1 Raw Materials

ACS (*Coffea arabica L*.) was obtained from Fazenda Yaguara Ecológica, situated in Taquaritinga do Norte, Pernambuco, Brazil (7°53′51.3″ S, 36°02′39.8″ W). The farm cultivates organically certified Arabica and Typica coffee varieties, which are roasted at 150°C for 20 min using a 10 kg‐capacity roaster. ICS (*C. arabica* L.) was supplied by São Braz S/A, located in Cabedelo, Paraíba, Brazil (7°05′07.1″ S, 34°51′01.9″ W), where coffee is processed under large‐scale roasting conditions at an average temperature of 210°C for 20 min in a 60 kg‐capacity roaster. ACS and ICS were the two specific CSS materials evaluated in this study, each obtained from a distinct source with different cultivation and processing histories.

The samples were milled in a ball mill to improve dispersion within the restructured salmon matrix, vacuum‐packed, and stored at −20°C until analysis. Laser diffraction analysis using a Beckman Coulter LS 13 320 (Brea, CA, USA) (Kang et al. 2019) showed a mean particle size of 43.7 µm. The particle size distribution (85% of particles between 8 and 128 µm) and representative optical microscopy images are presented in Table S1 and Figure S1, respectively.

### 2.2 Preparation of Restructured Salmon Products

Six whole salmons (*Salmo salar* L., originating from Norway) were acquired directly from a supplier in Cáceres, Spain. According to the traceability label, the fish was harvested approximately 5 days prior, corresponding strictly to the transit time from catch to our facilities, avoiding retail display exposure. During this period, the salmon was transported under refrigeration. The samples were processed immediately upon arrival at the IPROCAR laboratory. The six salmon were processed in three independent batches, with two salmon assigned to each batch. Within each batch, the meat from the two salmon was pooled before processing and formulation. Processing conditions were defined based on preliminary trials. The processing involved weighing the ingredients and grinding the salmon in a grinder (Bluesky model BMG 1300‐20) to simulate the collection of salmon trimmings for subsequent production of restructured nuggets. The ground salmon was then homogenized with salt (1%, w/w), microbial transglutaminase (Bindly, Girona, Spain; 0.215%, w/w), water (3.5%, w/w) in all formulations, and, when applicable, ICS or ACS (0.5%, w/w). Specifically, a CSS concentration of 0.5% (w/w) was selected on the basis of preliminary sensory evaluations and oxidative trials (Thiobarbituric acid reactive substances [TBARS]) to avoid excessive browning while ensuring antioxidant efficacy.

The mixture was molded into 4 × 4 × 3 cm^3^ molds, resulting in three treatments within each independent batch: a control (R‐C) without silverskin, an artisanal (R‐ACS), and an industrial (R‐ICS), each yielding 30 g pieces. The restructured pieces were allowed to rest for 24 h in a refrigerator at 3°C to enable transglutaminase activity. After resting, the pieces were cooked on a griddle (Repagas Model E64, Madrid, Spain) for 4 min on each side until reaching an internal temperature of 72°C ± 2°C. The precooked pieces were individually stored in polypropylene‐covered molds under aerobic conditions, protected from light, in a refrigerator at 3°C. For each independent batch, samples were randomly allocated across the five storage time points (1, 7, 14, 21, and 28 days) and analytical methods.

### 2.3 Physical and Chemical Characterization of CSS and Restructured Salmon

#### 2.3.1 Chemical Analysis

CSS samples (ACS and ICS) and restructured salmon products were characterized for moisture, ash, and protein content according to AOAC (2023). Lipid content was determined using the method described by Folch et al. (1957).

For both artisanal and ICS, total phenolic content (TPC) and phenolic composition were evaluated after hydroalcoholic extraction using an ethanol and ultrapure water mixture (1:1, v/v) at a sample‐to‐solvent ratio of 1:10 (w/v). Extraction was performed with 5 min of initial manual homogenization, followed by incubation in a water bath at 60°C for 60 min. The samples were then centrifuged at 3500 × *g* for 20 min at 10°C, followed by solvent removal under vacuum at 60°C. The extracts were prepared according to the validated protocol reported by Silva et al. (2020), with minor modifications. Subsequently, the dried residues obtained after rotary evaporation were resuspended in ultrapure water and adjusted to a final volume of 10 mL before analysis. Results were expressed on a sample weight basis.

The determination of TPC was performed using the Folin–Ciocalteu assay, following the procedure adapted from Abreu et al. (2024). Briefly, 10 µL of extract were mixed with 7990 µL of ultrapure water, 500 µL of Folin–Ciocalteu reagent (2 M), and 1500 µL of a 20% sodium carbonate solution. The reaction mixture was maintained in the absence of light for 2 h, after which absorbance was recorded at 765 nm at approximately 25°C. Phenolic content was expressed as milligrams of chlorogenic acid equivalents per 100 g of sample (mg CAE 100/g), using a calibration curve ranging from 10 to 50 µg/mL.

The phenolic profile was analyzed according to Padilha et al. (2017) using an Agilent 1260 Infinity LC system coupled to a diode array detector (DAD) (Agilent Technologies, Santa Clara, CA, USA). Separation was performed on a Zorbax Eclipse Plus RP‐C18 column (100 × 4.6 mm^2^, 3.5 µm), fitted with a Zorbax C18 guard column (12.6 × 4.6 mm^2^, 5 µm). A 20 µL aliquot was injected for each analysis. The mobile phase consisted of eluent A (phosphoric acid solution, pH 2.0) and eluent B (acidified methanol). Chromatographic separation was carried out at a flow rate of 0.8 mL/min under gradient conditions defined as follows: 0–5 min (5% B), 5–14 mi (23% B), 14–30 min (50% B), and 30–33 min (80% B). Data acquisition and processing were performed using OpenLAB CDS ChemStation software (Agilent Technologies).

The antioxidant capacity of the CSS (ACS and ICS) was determined using 2,2‐diphenyl‐1‐picrylhydrazyl (DPPH), 2,2′‐azino‐bis(3‐ethylbenzothiazoline‐6‐sulfonic acid) (ABTS), and ferric reducing antioxidant power (FRAP) assays.

Radical scavenging activity by DPPH was assessed by mixing 100 µL of extract with 3.9 mL of DPPH solution prepared in methanol. The reaction mixture was kept at 25°C for 30 min in the dark, and absorbance was recorded at 515 nm (Brand‐Williams et al. 1995). Quantification was performed using a Trolox calibration curve (50–1000 µM), and results were expressed as µmol TE 100/g of sample.

For the ABTS assay, 30 µL of extract was added to 3 mL of ABTS^+^ radical solution in methanol. After incubation for 6 min at 25°C, absorbance was measured at 734 nm against ethanol as blank (Re et al. 1999). Results were calculated as µmol TE 100/g of sample based on a Trolox standard curve (100–2000 µM).

FRAP was determined using a reagent composed of acetate buffer (300 mmol/L, pH 3.6), TPTZ solution (10 mmol/L in 40 mmol/L HCl), and FeCl_3_ (20 mmol/L) mixed in a 10:1:1 (v/v/v) ratio. A reaction mixture containing 90 µL of extract, 270 µL of water, and 2.7 mL of FRAP reagent was incubated at 37°C for 30 min. Absorbance was read at 595 nm, corresponding to the reduction of Fe^3+^ to Fe^2+^ (Benzie and Strain 1999). Results were expressed as µmol TE 100/g of sample using a Trolox calibration curve (50–1000 µM).

#### 2.3.2 Physical Evaluation of Restructured Salmon

Instrumental color evaluation was performed using a Minolta CR‐300 colorimeter (Konica Minolta, Osaka, Japan), equipped with illuminant D65, a 0° standard observer, and a 2.5 cm viewing port/area. Measurements followed the principles established by the Commission Internationale de L’Éclairage (CIE) (Billmeyer Jr. 1988). A total of five measurements were obtained for each sample group. The color coordinates evaluated were lightness (L*), redness (a*), and yellowness (b*).

Texture profile analysis (TPA) of the cooked restructured products was performed at room temperature using a TA‐XT2i Texture Analyser (Stable Micro Systems, Surrey, UK). Five square samples (4 × 4 × 3 cm^2^) were analyzed using a two‐cycle compression test. Samples were compressed to 40% of their original height with a 5 cm diameter cylindrical probe at a crosshead speed of 5 mm/s. Texture profile parameters were determined as described by Bourne (1978).

### 2.4 Oxidative Stability of Restructured Salmon

#### 2.4.1 Fatty Acid Profile

Fatty acid transesterification was conducted according to Sandler and Karo (1992). Lipids (10 mg) from R‐C, R‐ICS, and R‐ACS samples, previously extracted following Folch et al. (1957), were subjected to derivatization by adding 1 mL of sodium methoxide in methanol. The mixture was vortexed and incubated at 80°C for 30 min in a dry oven. Subsequently, 1 mL of sulfuric acid in methanol was added, and the samples were vortexed and incubated again at 80°C for 30 min.

After cooling to room temperature, 1 mL of *n*‐hexane and 1 mL of saturated aqueous sodium chloride solution were added. The mixture was vortexed and centrifuged, and the upper organic phase containing fatty acid methyl esters (FAMEs) was recovered using a Pasteur pipette and transferred to a 2 mL GC vial. The solvent was evaporated under a nitrogen stream, and the residue was reconstituted in 1 mL of *n*‐hexane.

FAMEs were analyzed by gas chromatography using a Hewlett–Packard HP‐5890A gas chromatograph equipped with a flame ionization detector (GC–FID). Separation was performed on a cyanopropyl capillary column (ZEBRON ZB‐171 FAME, Phenomenex, CA, USA; 20 m × 0.18 mm i.d.; 0.15 µm film thickness). Injector and detector temperatures were maintained at 250°C.

The oven temperature program started at 150°C, increased at 10°C/min to 180°C (held for 1 min), and then increased at 7°C/min to 205°C, where it was held for 2 min. Quantification was performed using a calibration curve constructed with a commercial FAME standard mixture (Supelco 37 Component FAME Mix, Merck), and results were expressed as percentage of total FAMEs in the sample.

#### 2.4.2 TBARS Determination

TBARS were determined according to Ganhão et al. (2011), with slight modifications. Briefly, 5 g of sample were transferred to Falcon tubes and homogenized with 15 mL of 3.86% perchloric acid. During homogenization, the tubes were kept in an ice bath to minimize oxidative reactions. The homogenate was filtered and centrifuged at 3000 rpm for 4 min. Then, 2 mL of the supernatant were mixed with 2 mL of 0.02 M thiobarbituric acid solution in test tubes. The mixtures were heated at 90°C for 45 min in an oven, together with standard curve tubes. After cooling, absorbance was measured at 532 nm. The standard curve was prepared using 1,1,3,3‐tetraethoxypropane (TEP) (0.2268 g) dissolved in 3.86% perchloric acid. Results were expressed as mg of malondialdehyde (MDA) per kg of sample.

#### 2.4.3 Carbonyl Compound Quantification

2,4‐dinitrophenylhydrazine (DNPH)‐reactive carbonyl content was determined through derivatization with DNPH following Estévez et al. (2005), with slight modifications. For the assay, 1 g of restructured sample was minced and homogenized (1:10, w/v) in 20 mM sodium phosphate buffer containing 0.6 M NaCl (pH 6.5), using an Ultra‐Turrax homogenizer (IKA‐Werke GmbH & Co. KG, Staufen, Germany) for 40 s.

The resulting homogenate was split into two equal portions (0.2 mL each) and transferred to 2 mL microtubes. Protein precipitation was achieved by adding 1 mL of ice‐cold 10% (w/v) trichloroacetic acid (TCA), followed by centrifugation for 5 min at 5000 rpm. After discarding the supernatant, the first pellet was incubated with 1 mL of 2 M HCl for protein determination, whereas the second pellet was reacted with an equal volume of 0.2% (w/v) DNPH prepared in 2 M HCl for carbonyl derivatization.

Both reaction systems were maintained at room temperature for 1 h. Subsequently, proteins were reprecipitated using 1 mL of 10% TCA and washed twice with a mixture of ethanol and ethyl acetate (1:1, v/v) to eliminate excess DNPH. The cleaned pellets were solubilized in 1.5 mL of 20 mM sodium phosphate buffer containing 6 M guanidine hydrochloride (pH 6.5), vortexed, and centrifuged for 2 min at 5000 rpm to remove insoluble residues.

The spectrophotometer was zeroed using the 6 M guanidine hydrochloride solubilization buffer. Protein content was quantified spectrophotometrically at 280 nm using the HCl tube as the formulation‐specific sample blank and bovine serum albumin (BSA) as the calibration standard. The absorbance of the DNPH‐treated sample was measured at 370 nm, and the absorbance of the corresponding formulation‐specific HCl tube was subtracted to obtain the net absorbance. Carbonyl levels were expressed as nmol of DNPH‐reactive carbonyls per mg of protein, based on a molar extinction coefficient of 21.0 mM^−1^/cm.

#### 2.4.4 Volatile Compounds

Minced salmon samples (5 g) were transferred into 20 mL headspace vials and spiked with 0.4 µg of cyclohexanone, used as internal standard. Prior to extraction, the vials were equilibrated for 30 min at 50°C to promote volatile release. Volatile compounds were subsequently isolated by solid‐phase microextraction (SPME) using a DVB/CAR/PDMS fiber (Supelco, Bellefonte, PA, USA) exposed to the headspace for 30 min under controlled conditions. Desorption of analytes was performed in the GC injection port at 250°C for 5 min in splitless injection mode. Chromatographic analysis was carried out on an Agilent 7890B gas chromatograph coupled to a 5977A mass selective detector (Agilent Technologies, Palo Alto, CA, USA). High‐purity helium was used as the carrier gas at a constant flow rate of 1.2 mL/min. Separation of volatile fractions was achieved using two capillary columns with different polarities: a nonpolar HP‐5 MS column and a polar VF‐WAXms column (both 30 m × 0.25 mm i.d., 0.25 µm film thickness; Agilent Technologies). The oven temperature program was initiated at 35°C (held for 3 min), followed by a linear increase to 250°C at 5°C/min, with a final holding period of 10 min. Mass spectrometric detection was performed in electron impact mode with a scan range of 35–500 amu and a data acquisition rate of 50 Hz, whereas the ion source was maintained at 230°C. Volatile compounds were analyzed using three independent biological batches (*n* = 3). The results are reported as integrated peak areas. Compounds not detected were designated as non‐detected (ND) and assigned a value of zero for subsequent statistical and chemometric analyses. Volatile identification was based on comparison of mass spectra with NIST14 and Wiley libraries, combined with calculated retention indices obtained from a homologous series of *n*‐alkanes (C_7_–C_30_).

### 2.5 Microbiological Analysis

Microbiological safety of the restructured salmon was assessed prior to sensory testing to ensure suitability of the samples throughout refrigerated storage. The analysis comprised enumeration of aerobic mesophilic microorganisms on Plate Count Agar (PCA), incubated at 30°C for 24 h, and quantification of Enterobacteriaceae on Violet Red Bile Glucose Agar (VRBG) incubated at 37°C for 24 h, and enumeration of lactic acid bacteria (LAB) on Man, Rogosa, and Sharpe (MRS) agar, incubated at 30°C for 48 h under microaerophilic conditions by enclosing the plates in a plastic bag to create a reduced‐oxygen environment. All the microbiological analyses were performed using the standard plate count approach.

In addition, *Listeria monocytogenes* detection was carried out according to ISO 11290‐1, with results reported as presence or absence in 25 g of sample. Microbial loads were expressed as log CFU/g, except for *L. monocytogenes*. Only samples meeting the acceptability threshold of <2 log CFU/g for Enterobacteriaceae were selected for sensory evaluation (Table S3 **;** Indio et al. 2024) to support the microbiological suitability of the samples during the panel sessions.

### 2.6 Sensory Analysis

The sensory analysis of the restructured salmon products was conducted following ethical approval granted by the Delegation of the Bioethics and Biosafety Commission of the University of Extremadura (approval number 209/2024), in accordance with the 1964 Declaration of Helsinki and its later amendments or equivalent ethical standards. Informed consent was obtained from all participants prior to their involvement in the study.

The products were evaluated by a trained panel of 18 assessors with extensive experience in the sensory descriptive analysis of animal‐derived foods. Panelists were trained in the identification and evaluation of the sensory descriptors used in the study, including those associated with oxidative deterioration, such as rancidity and warmed‐over flavor (WOF). The evaluated attributes included pink color, fishy odor, juiciness, fresh fish flavor, fibrousness, rancidity, WOF, saltiness, sourness, and bitterness, with particular attention to oxidation‐related perceptions. Sensory evaluations were conducted in individual booths at an ambient temperature of 22°C ± 2°C under standardized white daylight illumination. Trained‐panel liking was also evaluated.

For the experimental phase, samples were removed from refrigeration and briefly heated in a microwave oven at maximum power (800 W) for 10–15 s until reaching an internal temperature of 65°C ± 2°C (measured with a digital probe thermometer) to ensure uniform preparation. Subsequently, 30 g portions were served on white plates labeled with randomly generated three‐digit codes and presented in randomized order to minimize positional bias. Each attribute was evaluated using a 10 cm unstructured line scale, anchored at 0 (absence/low intensity; light color, where applicable) and 10 (maximum intensity; dark color, where applicable). Mineral water and fresh apple slices were provided between samples for palate cleansing. Mean scores were calculated for each descriptor across independent replicates.

### 2.7 Statistical Analysis

The experimental unit was the independent batch of restructured salmon, and the experiment was performed in triplicate (*n* = 3). Within each batch, three technical replicates were used for chemical and oxidative assays, five measurements were taken for physical analyses (color and texture), and 18 assessors were involved in sensory evaluation. Data are presented as mean ± standard deviation.

Differences between CSS samples (ACS vs. ICS) were evaluated using Student's *t*‐test. Data from restructured salmon were analyzed using two‐way ANOVA, with treatment and storage time as fixed factors. For sensory data, assessor was included as a random factor to account for panel variability. When significant effects were detected (*p* < 0.05), means were compared using Tukey's post hoc test. Statistical analyses were performed using XLSTAT software (version 2014.5.03, Addinsoft, USA).

### 2.8 Preparation and Data Visualization

For chemometric analysis of volatile compounds, a heat map combined with hierarchical cluster analysis (HCA) was generated using TBtools‐II v2.105 (Chen et al. 2023) and OriginPro 2024. Prior to analysis, volatile data were preprocessed by autoscaling, consisting of subtracting the mean of each variable and dividing by its standard deviation.

In HCA, Euclidean distance was used as the similarity metric, and clustering was performed using the group average (UPGMA) method. Additionally, principal component analysis (PCA‐Biplot) was applied to the sensory dataset across storage time using XLSTAT software.

## 3 Results and Discussion

### 3.1 Chemical and Bioactive Characterization of ICS and ACS

The chemical characteristics of ICS and ACS (*C. arabica* L.) were analyzed, revealing similar values for protein (ACS: 14.21% ± 0.42%, ICS: 14.69% ± 0.12%), lipids (ACS: 9.24% ± 1.44%, ICS: 8.19% ± 1.50%), and ash (ACS: 4.83% ± 0.35%, ICS: 4.50% ± 0.11%). However, the by‐products moisture content exhibited a statistically significant difference, at 5.27% ± 0.11% and 3.32% ± 0.25%, respectively.

In contrast, Ateş and Elmacı (2019) reported higher levels of moisture (5.42%), protein (15.36%), and ash (7.64%), as well as lower lipid content (4.61%). These variations can be attributed to coffee cultivation conditions, such as geographical origin, climate, processing, and storage. These factors reflect the distinction in the physicochemical properties of these by‐products (Tieghi et al. 2024).

The characterization of antioxidant compounds (Table 1) revealed that the ACS sample exhibited a higher concentration of total phenolic compounds (TPC) and superior antioxidant activity in the DPPH and ABTS assays. In contrast, the ICS sample demonstrated greater reducing capacity, as evidenced by its significantly higher FRAP assay values.

**TABLE 1:** Composition of antioxidant compounds and antioxidant capacity of artisanal and industrial coffee silverskins (*Coffea arabica*).

Composition	ICS	ACS	*p* value
Total Phenolic Compounds (TPC)*	2311.93 ± 9.7	2662.99 ± 87.99	0.0035
DPPH **	302.82 ± 15.85	871 ± 4.90	<0.0001
ABTS **	754.75 ± 18.56	1536.23 ± 16.71	<0.0001
FRAP **	14,174.48 ± 31.95	5803.98 ± 53.25	<0.0001
**Phenolic Compounds (mg**/**100 g)**			
Gallic acid	1.095 ± 0.007	1.288 ± 0.073	0.003
Vanillic acid	1.647 ± 0.008	1.481 ± 0.010	<0.0001
Syringic acid	0.977 ± 0.003	1.026 ± 0.011	0.0004
Naringin	16.427 ± 0.016	53.081 ± 1.163	<0.0001
Chlorogenic acid	9.202 ± 0.091	257.487 ± 1.294	<0.0001
Caffeic acid	ND	1.088 ± 0.005	<0.0001
*p*‐coumaric acid	1.416 ± 0.063	15.082 ± 0.068	<0.0001
Ferulic acid	ND	0.794 ± 0.011	<0.0001
Quercetin hydrate	ND	1.581 ± 0.038	<0.0001

*Note*: Values are means ± standard deviations (*n* = 3). *TPC results are expressed as mg CAE·100/g of sample; ** DPPH, ABTS, and FRAP results are expressed as µmol TE 100/g of sample.

Abbreviations: ACS, artisanal coffee silverskin; DPPH, 2,2‐diphenyl‐1‐picrylhydrazyl; FRAP, ferric reducing antioxidant power; ICS, industrial coffee silverskin; ND, not detected.

These differences reflect the distinct mechanisms underlying the antioxidant assays: Although DPPH and ABTS evaluate free radical‐scavenging capacity, the FRAP assay measures electron‐donating ability through the reduction of Fe^3+^ to Fe^2+^ ions (Dudonné et al. 2009). These findings are consistent with the results reported by Vignoli et al. (2011), who associated DPPH radical‐scavenging activity with the presence of phenolic compounds. They also align with the study by Li et al. (2024), which indicates that more intense thermal processes, such as industrial roasting, promote Maillard reactions and the formation of non‐phenolic compounds with high reducing power, including high‐molecular‐weight melanoidins. Furthermore, as recently elucidated by Bolchini et al. (2025), advanced Maillard reactions generate specific reductone structures (e.g., DDMP) that act as electron donors, supporting an electron‐transfer‐based antioxidant mechanism and potentially explaining the higher FRAP values observed in the industrial sample. Overall, the antioxidant capacity of CSS likely results from synergistic interactions among phenolic compounds, reductones, and melanoidins; however, the roasting profile modulates the predominance of these mechanisms, shifting from radical‐scavenging pathways under milder conditions to electron‐transfer‐driven responses with more intense thermal processing.

Therefore, roasting conditions directly influence the phenolic profile and the expression of antioxidant capacity due to the complexity of the chemical reactions involved in the roasting process (Muñoz et al. 2020).

Regarding individual phenolic compounds, ACS exhibited a markedly higher concentration of chlorogenic acid (257.49 mg/100 g) than ICS (9.20 mg/100 g). The ACS sample also showed elevated levels of naringin (53.08 mg/100 g vs. 16.43 mg/100 g) and *p*‐coumaric acid (15.08 mg/100 g vs. 1.42 mg/100 g). Additionally, caffeic acid (1.09 mg/100 g), ferulic acid (0.79 mg/100 g), and quercetin (1.58 mg/100 g) were detected exclusively in ACS, underscoring its greater phenolic diversity and structural complexity. Conversely, the ICS sample contained slightly higher levels of vanillic acid (1.65 mg/100 g vs. 1.48 mg/100 g), which may reflect differences in the specific sources, cultivars, and processing histories of the two samples. This pattern may also be associated with the degradation of more complex, heat‐sensitive phenolic structures into smaller molecular fragments during thermal processing (Alcantara et al. 2025).

Coffee roasting constitutes a key thermal processing operation characterized by extensive chemical changes within the bean matrix. Among the most relevant transformations are Maillard reactions, caramelization processes, and the thermal degradation of chlorogenic acids, their isomeric forms, and other phenolic constituents (Kašpar et al. 2025). These reactions reshape the redox profile of CSS by altering the balance between phenolics and Maillard‐derived compounds. Consequently, roasting is a primary factor that not only modulates the overall antioxidant capacity of CSS but may also shift the predominant antioxidant mechanism, reorienting the system from radical‐scavenging pathways toward electron‐transfer‐driven responses, as reflected in in vitro assays (Muñoz et al. 2020; Li et al. 2024; Bolchini et al. 2025). However, it must be acknowledged that the differences observed between ACS and ICS cannot be attributed solely to roasting severity, as differences in origin, cultivar, and post‐harvest practices represent additional sources of variation that may have contributed to the distinct chemical profiles observed (Tieghi et al. 2024).

### 3.2 Characterization of Restructured Salmon Products

As shown in Table 2, the physicochemical composition of the restructured salmon indicates that the formulations containing CSS (both artisanal and industrial) had similar values for pH, total protein and mineral content compared to the control sample. However, these formulations exhibited reduced moisture and lipid content.

**TABLE 2:** Chemical and physical characterization of restructured salmon.

Parameters	Restructured salmon		
	R‐C	R‐ICS	R‐ACS
**Composition**			
pH	6.31 ± 0.04^a^	6.23 ± 0.06^a^	6.22 ± 0.07^a^
Moisture	64.08 ± 0.72^a^	61.73 ± 0.25^b^	62.11 ± 0.94^b^
Protein	19.1 ± 1.3^a^	18.9 ± 0.7^a^	20.7 ± 0.8^a^
Lipid	9.33 ± 0.32^a^	7.38 ± 0.10^b^	7.35 ± 0.17^b^
Ash	2.18 ± 0.08^a^	2.20 ± 0.08^a^	2.25 ± 0.05^a^
**Texture profile**			
Hardness (N)	74.6 ± 5.5^a^	77.6 ± 8.2^a^	83.3 ± 6.4^a^
Adhesiveness (N s)	−0.1 ± 0.0^a^	−0.2 ± 0.1^a^	−0.2 ± 0.2^a^
Springiness	0.78 ± 0.03^a^	0.79 ± 0.02^a^	0.79 ± 0.03^a^
Cohesiveness	0.63 ± 0.07^a^	0.63 ± 0.07^a^	0.63 ± 0.03^a^
Gumminess (N)	47.1 ± 6.1^a^	49.0 ± 7.3^a^	52.4 ± 7.2^a^
Chewiness (N)	36.9 ± 5.9^a^	38.8 ± 6.3^a^	41.7 ± 6.8^a^
Resilience	0.24 ± 0.05^a^	0.24 ± 0.04^a^	0.24 ± 0.04^a^
**Fatty acids (% total FAMEs)**			
C14:0	1.87 ± 0.11^a^	2.03 ± 0.01^a^	1.99 ± 0.03^a^
C16:0	9.96 ± 0.48^a^	10.28 ± 0.61^a^	10.37 ± 0.24^a^
C16:1*n*‐7	2.38 ± 0.20^a^	2.05 ± 0.01^b^	2.06 ± 0.08^b^
C18:0	2.81 ± 0.16^a^	2.94 ± 0.20^a^	2.93 ± 0.07^a^
C18:1*n*‐9c	49.24 ± 1.73^a^	47.85 ± 0.58^a^	47.47 ± 0.20^a^
C18:2*n*‐6c	16.41 ± 0.56^b^	17.33 ± 0.20^a^	17.27 ± 0.03^ab^
C18:3*n*‐3	5.51 ± 0.02^b^	5.63 ± 0.04^a^	5.58 ± 0.02^a^
C20:1*n*‐9	2.91 ± 0.06^a^	2.86 ± 0.03^a^	2.88 ± 0.05^a^
C20:2*n*‐6	1.26 ± 0.06^a^	ND^b^	ND^b^
C20:5*n*‐3 (EPA)	1.85 ± 0.09^b^	2.08 ± 0.03^a^	2.11 ± 0.04^a^
C22:6*n*‐3 (DHA)	5.80 ± 0.54^b^	6.95 ± 0.06^a^	7.34 ± 0.11^a^

*Note*: R‐C = control restructured salmon (no antioxidants); R‐ICS = restructured salmon with industrial coffee silverskin; R‐ACS = restructured salmon with artisanal coffee silverskin. Values are means ± standard deviations (*n* = 3). Different lowercase letters within the same row indicate significant differences (*p* < 0.05) between samples.

Abbreviations: DHA, docosahexaenoic acid; EPA, eicosapentaenoic acid; FAME, fatty acid methyl esters; ND, not detected.

Adding dry ingredients, such as CSS, to high‐moisture matrices, such as fresh fish meat products, can reduce the final product's moisture content. This is due to the hygroscopic characteristics of silverskin, which promote water absorption and redistribution of moisture within the matrix (Martuscelli et al. 2021). From microbiological and oxidative standpoints, this reduction can be beneficial, as products with lower water activity tend to be more stable (Mafe et al. 2024).

Regarding lipid content, samples with added silverskin (R‐ICS and R‐ACS) exhibited lower values than the control sample (R‐C), which is consistent with previous studies. Sánchez‐Alonso et al. (2011) found similar results in minced mackerel muscle samples with added wheat dietary fiber and caffeic acid. Furthermore, considering the inherent lipid content of the added CSS, the observed reduction may also reflect differences in lipid extraction efficiency during analysis, potentially associated with interactions among phenolic compounds, proteins, dietary fibers, and lipids within the matrix.

The analysis of individual fatty acids revealed changes in their relative profile following CSS incorporation. Notably, palmitoleic acid (C16:1) decreased, and eicosadienoic acid (C20:2) was not detected in CSS‐treated formulations compared to the control sample (R‐C). Long‐chain polyunsaturated fatty acids, such as eicosapentaenoic acid (EPA, C20:5*n*‐3) and docosahexaenoic acid (DHA, C22:6*n*‐3), exhibited significantly higher relative proportions in CSS‐treated samples (R‐ICS and R‐ACS) than in the control (R‐C) (*p* < 0.05). These findings indicate that CSS incorporation was associated with higher relative proportions of EPA and DHA in the restructured salmon after thermal processing. This result is consistent with previous reports showing that plant‐derived antioxidants may contribute to maintaining omega‐3 fatty acid levels during thermal processing of salmon‐based products (R. Ferreira et al. 2022; Gouvêa et al. 2023).

From a technological standpoint, the changes in physicochemical parameters were insufficient to significantly impact the texture attributes of the samples (*p* > 0.05; Table 2). This suggests that adding CSS does not compromise the structural standardization among formulations, which is desirable in processed products.

Regarding the color of the samples, presented in Figure 1, the incorporation of CSS, both industrial and artisanal, significantly darkened the restructured salmon, evidenced by the decrease in lightness (L*) values compared to the control sample. This change is associated with the dark color of the silverskin, resulting from the formation of melanoidins during the coffee roasting process (Chemat et al. 2024).

**FIGURE 1:**
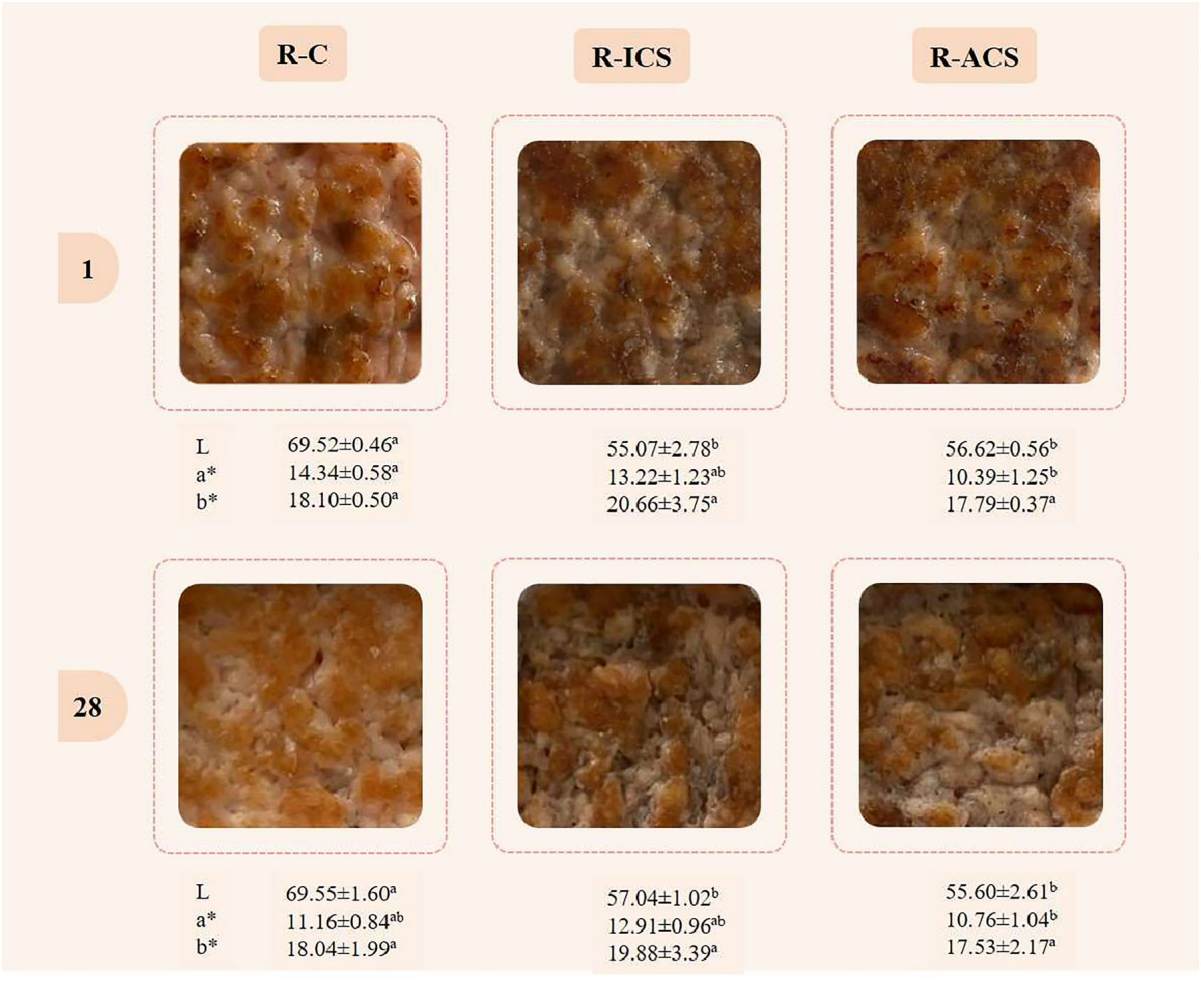
Color of restructured salmon products during storage. R‐C = control restructured salmon (no antioxidants); R‐ICS = restructured salmon with industrial coffee silverskin; R‐ACS = restructured salmon with artisanal coffee silverskin. Values are mean ± standard deviation (*n* = 5). Different lowercase letters within the same row indicate significant differences (*p* < 0.05) between samples.

When comparing the initial and final storage points (Days 1 and 28), treatments with CSS exhibited less variation in the a* parameter, indicating an overall stabilization of the reddish coloration in these samples. In contrast, the control sample demonstrated a more pronounced decrease in this parameter by the end of the 28‐day period, suggesting pigment degradation. The observed stability in the treated samples can be attributed to the presence of phenolic compounds, particularly chlorogenic acid derivatives (Zhang et al. 2024), although a more cautious interpretation is needed as specific pigment or carotenoid data would be required to confirm the exact stabilization mechanism.

Phenolic compounds act as natural antioxidants by inhibiting oxidative reactions responsible for color loss. The pigmentation of salmon is predominantly attributable to carotenoids, such as astaxanthin and canthaxanthin, which exhibit sensitivity to oxidation. The observed overall decrease in the a* value in the control sample (R‐C) suggests potential depletion of these pigments by the end of the assay (Grünenwald et al. 2019). No significant changes were observed in the b* values between the beginning and the end of the storage in any treatment, indicating that the addition of CSS and refrigerated storage did not perceptibly affect the yellowish hue of the restructured salmon products.

### 3.3 Lipid Oxidation and DNPH‐Reactive Carbonyls in Restructured Salmon

Salmon is particularly susceptible to lipid oxidation due to its high content of polyunsaturated fatty acids. This process has been widely studied due to its detrimental effects on sensory and nutritional quality, mainly associated with the formation of undesirable compounds, such as MDA (Grünenwald et al. 2019).

The incorporation of CSS resulted in a pronounced reduction in MDA levels in restructured salmon samples throughout the 28‐day refrigerated storage period, highlighting its effectiveness in controlling lipid oxidation (Figure 2A). Both R‐ICS and R‐ACS treatments exhibited consistently lower MDA concentrations compared to the control at all evaluated time points, with only minor variations during storage (*p* < 0.05). In the R‐ACS samples, MDA values ranged from 0.313 mg/kg on Day 1 to 0.376 mg/kg on Day 28, with a slight increase observed on Day 21 (0.454 mg/kg). Similarly, the R‐ICS treatment showed MDA levels varying between 0.337 and 0.480 mg/kg, with the highest value recorded on Day 14. In contrast, the control samples (R‐C) showed a significant and progressive increase in lipid oxidation (*p* < 0.05), with MDA values rising from 1.894 mg/kg on Day 1 to a peak of 3.927 mg/kg on Day 21, followed by a slight reduction on Day 28 (3.466 mg/kg). These results demonstrate the protective effect of CSS against lipid oxidation in polyunsaturated fatty acid‐rich matrices such as salmon.

**FIGURE 2:**
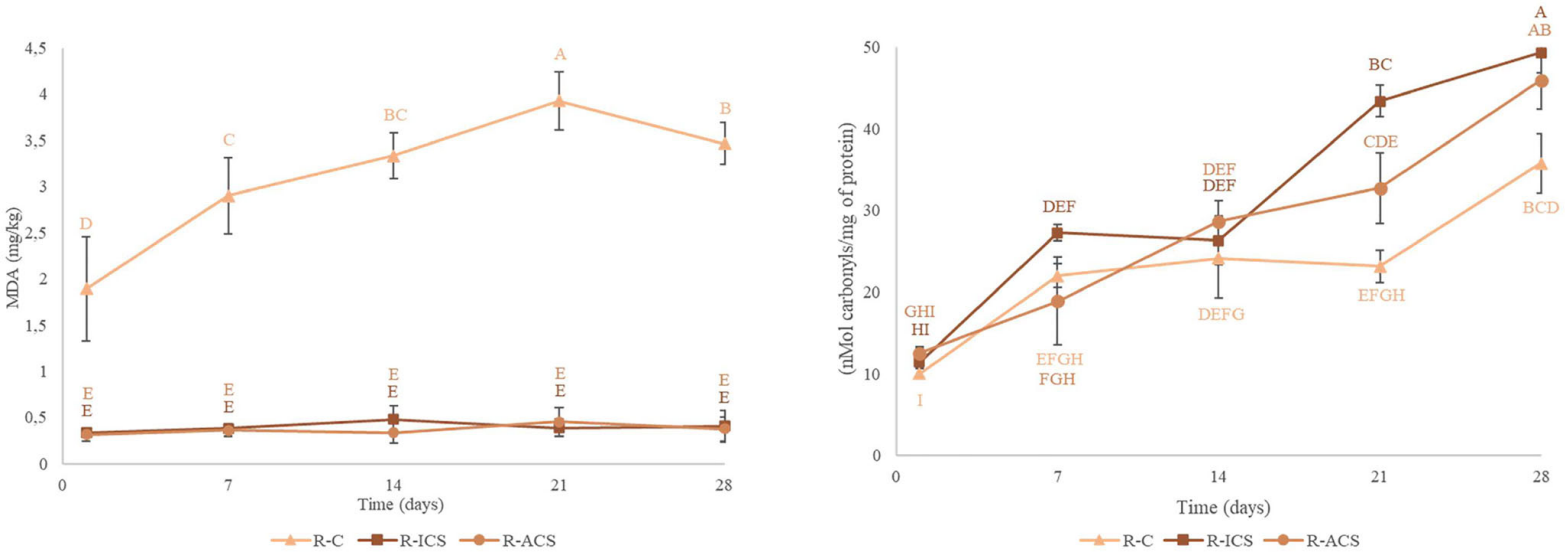
Oxidation results for restructured salmon products during storage. (A) TBARS values (mg MDA/kg sample); (B) carbonyl content (nmol carbonyl/mg protein). R‐C = control restructured salmon; R‐ICS = restructured salmon with industrial coffee silverskin; R‐ACS = restructured salmon with artisanal coffee silverskin. Results are expressed as mean ± standard deviation. Different lowercase letters indicate significant differences (*p* < 0.05) between samples at the same storage time; Different uppercase letters indicate significant differences (*p* < 0.05) within the same sample over storage time. MDA, malondialdehyde.

Previous studies have demonstrated antioxidant effects in salmon burgers enriched with *Salvia officinalis* (0.5%–1.5%), presenting TBARS values between 0.09 and 0.27 mg MDA/kg after frozen storage (R. Ferreira et al. 2022). Similarly, Cilli et al. (2020) reported reduced MDA formation in salmon burgers supplemented with grape pomace flour (1%–2%). In the present study, the addition of a relatively low concentration (0.5%) of CSS effectively maintained oxidative stability, reducing MDA levels even after cooking and refrigerated storage, conditions known to accelerate oxidative reactions, thereby supporting its potential as a natural antioxidant ingredient for perishable fish products.

Martuscelli et al. (2021) and Vargas‐Sánchez et al. (2024) reported that CSS effectively limited lipid oxidation in both raw and grilled chicken burgers and in pork meat homogenates, respectively. Extending these findings, Mesárošová et al. (2025) demonstrated that the application of both CSS extracts and powders in Frankfurter‐type sausages effectively hindered lipid oxidation. The authors reported that the MDA content in CSS‐treated sausages was restricted to 0.14 mg/kg, showing values comparable to those obtained with ascorbic acid treatment and remaining substantially below the untreated control group. These results highlight the potential of CSS as a clean‐label antioxidant ingredient for controlling lipid oxidation in different food matrices, whereas further studies are needed to determine the performance of CSS from diverse sources and processing conditions.

The stability of fish products during storage depends largely on the equilibrium between oxidative reactions and the antioxidant capacity of the food matrix (Gouvêa et al. 2023). In the present study, the superior oxidative stability observed in the treated samples suggests distinct antioxidant mechanisms associated with the two silverskin sources. For the R‐ACS samples, this protection may be associated primarily with the rich phenolic and alkaloid profile, including chlorogenic acid and caffeine, which are known to neutralize reactive species (•OH and ROO•) and chelate prooxidant metal ions (Costa et al. 2018). Conversely, despite the extensive thermal degradation of chlorogenic acids, the R‐ICS treatment proved equally effective. This suggests that the advanced Maillard reaction products generated during industrial roasting, including reductones and high‐molecular‐weight melanoidins, may contribute as electron‐donating compounds within the lipid matrix (Bolchini et al. 2025; Li et al. 2024). However, antioxidant efficacy is influenced by multiple factors, including processing effects, the ratio between antioxidants and susceptible lipids, and, most importantly, the ability of these bioactive molecules to access vulnerable sites prone to oxidative degradation (Sánchez‐Alonso et al. 2011; Pateiro et al. 2021).

Although in vitro assays provide valuable insights into antioxidant potential, their performance can vary substantially in complex food matrices, such as restructured salmon. This variability may explain the similar TBARS behavior between samples containing artisanal (ACS) and industrial (ICS) silverskin, despite the potentially higher in vitro antioxidant activity of ACS (Sánchez‐Alonso et al. 2011).

The findings demonstrate a protective effect of CSS (0.5%) against secondary lipid oxidation in restructured salmon products during refrigerated storage, suggesting beneficial activity of both ACS and ICS sources. However, the specific mechanisms of action of these sources may vary according to extract origin and composition.

In contrast to the lipid protection observed, the evaluation of DNPH‐reactive carbonyls (Figure 2B) revealed a different dynamic. Although the untreated control (R‐C) displayed a steady, progressive increase in these values throughout refrigerated storage, the samples treated with CSS (R‐ACS and R‐ICS) experienced a more pronounced accumulation of apparent carbonyls. Notably, the values in R‐ACS and R‐ICS were statistically higher than those in the control sample at the later storage time points, indicating that silverskin incorporation was associated with higher apparent carbonyl levels under the conditions evaluated. These findings should, therefore, be interpreted cautiously, as they may represent a potentially adverse outcome for the protein fraction.

Although both artisanal and industrial silverskin treatments influenced DNPH‐reactive carbonyl levels, the underlying mechanisms cannot be clearly distinguished from those involved in lipid degradation. The observed variability may be associated with the potential prooxidant behavior of certain phenolic matrices under specific redox conditions, depending on antioxidant dose, protein structure, and interactions within the food matrix (Estévez 2021; Günal‐Köroğlu et al. 2025). Although secondary lipid oxidation products (e.g., MDA and reactive aldehydes) typically react with amino groups to form Schiff bases and nonprotein carbonyl derivatives (Estévez 2011), the strongly reduced MDA levels in CSS‐treated samples suggest that lipid‐derived carbonyl formation alone may not explain the observed differences. In complex multiphase food systems, phenolic compounds can be oxidized into electrophilic quinones that covalently bind to muscle proteins. Therefore, the higher apparent carbonyl levels in CSS‐treated samples may not exclusively reflect direct oxidative cleavage and could, at least partially, be influenced by matrix‐related effects or other analytical interferences. Thus, phenol–protein interactions and potential matrix effects may contribute to higher apparent carbonyl levels even when lipid oxidation is effectively controlled (Estévez 2021).

These data indicate that, although CSS successfully reduced lipid oxidation, CSS‐treated samples showed higher apparent DNPH‐reactive carbonyl levels at the tested dose (0.5%). Furthermore, the distinction between industrial and artisanal silverskin underscores the notion that compositional variations may influence their antioxidant or prooxidant behavior (Costa et al. 2018). It is noteworthy that there is a paucity of literature addressing the specific impact of CSS on DNPH‐reactive carbonyl formation in fish products.

### 3.4 Volatile Compound Composition of Restructured Salmon During Storage

The volatile compounds identified in the restructured salmon (R‐C, R‐ICS, and R‐ACS) are shown in Figure 3, evaluated at 1, 14, and 28 days of refrigerated storage. A total of 22 compounds were detected and represented in a heat map with hierarchical clustering, covering aldehydes, ketones, alcohols, furans, and terpenes.

**FIGURE 3:**
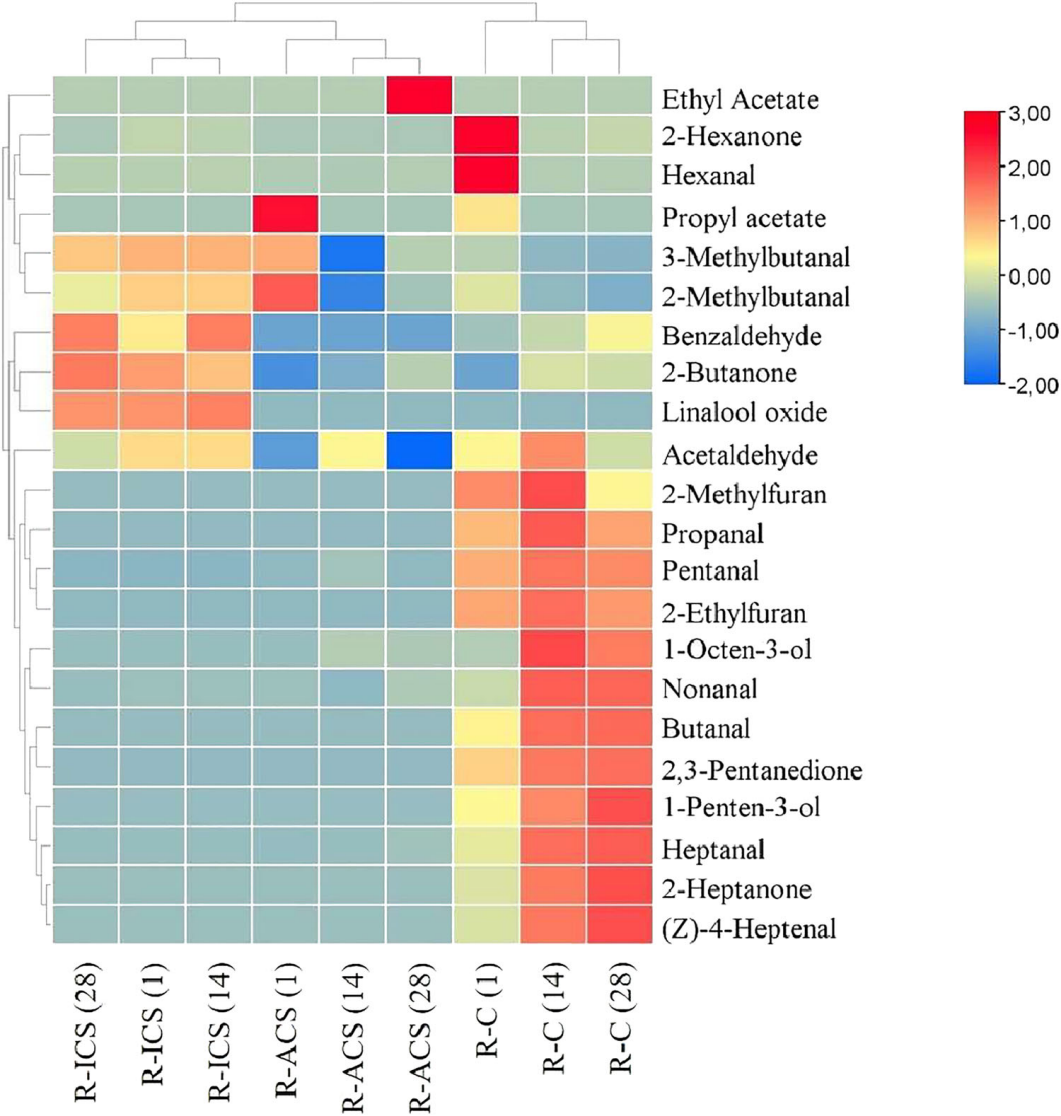
Heatmap with hierarchical clustering applied to the volatile compound profile of restructured salmon during storage. R‐C = control restructured salmon; R‐ICS = restructured salmon with industrial coffee silverskin; R‐ACS = restructured salmon with artisanal coffee silverskin.

Regarding sample clustering, three well‐defined clusters are observed, organized mainly according to the formulations. This exploratory visualization, supported by the semiquantitative analysis (Table S2), suggests that the presence or absence of CSS contributed to the observed grouping of volatile profiles, together with the effect of storage time. Although extended storage favored the formation of volatile compounds associated with lipid oxidation, this behavior was markedly more pronounced in the control sample without added natural antioxidants. This outcome underscores the potential antioxidant capacity of CSS in maintaining the quality of fish meat products.

Notably, the formulation R‐ACS exhibited a reduced relative abundance of oxidative volatile compounds on Days 14 and 28, suggesting a potential protective effect against oxidative deterioration. This observation suggests a potential enhancement in the inhibition of oxidation during storage, which may be attributable to an elevated concentration or distinct profile of phenolic compounds present in the artisanal sample (ACS), as demonstrated in Table S2.

Specifically, volatile aldehydes constituted the predominant class among the identified compounds. The control formulation (R‐C) exhibited the highest levels of these compounds throughout the storage period, followed by the formulation with industrial silverskin (R‐ICS) and, to a lesser extent, the formulation with artisanal silverskin (R‐ACS), as demonstrated in Table S2.

The formation of these aldehydes can be initiated during heat treatment, contributing positively to the development of grilled aroma. However, during storage, their presence is directly associated with oxidative degradation of lipids, resulting in rancid notes and undesirable flavors (de F. Marques et al. 2022).

Among the aldehydes commonly associated with the development of undesirable flavors in meat products and seafood, those derived from lipid oxidation, such as hexanal, pentanal, heptanal, nonanal, and (*Z*)‐4‐heptenal, stand out as sensory markers of rancidity (Ortuño et al. 2021; Qi et al. 2021; Dai et al. 2024).

The greater abundance of lipid‐derived volatile compounds in the R‐C sample is consistent with a higher susceptibility to oxidation, which underlines the importance of adding antioxidants to fish‐based products. In this context, adding CSS, regardless of its processing history (ACS or ICS), was associated with lower levels of volatile markers of lipid oxidation and rancidity. This suppression of off‐flavor precursors is consistent with the TBARS results discussed previously, emphasizing the potential of CSS to mitigate sensory deterioration in complex matrices.

### 3.5 Sensory Analysis

The microbiological results supported restricting the sensory evaluation to 14 days of refrigerated storage. No *L. monocytogenes* was detected in any of the analyzed samples. Aerobic mesophilic counts remained below 3 log CFU/g in every sampled batch on Days 1, 7, 14, and 21. Concomitantly, LAB counts remained below the detection limit (<2 log CFU/g) in all formulations throughout refrigerated storage (Table S3), suggesting that no detectable spontaneous lactic fermentation occurred during storage. In contrast, Enterobacteriaceae were not detected during the first 14 days. On Day 21, these microorganisms were detected only in the R‐ICS formulation, at 2.63 ± 1.22 log CFU/g. This isolated detection was considered with caution because of the relatively high variability observed. Therefore, sensory evaluation was conducted on Days 1, 7, and 14. This 14‐day period was used to define the storage duration considered for sensory evaluation and does not represent a validated commercial shelf life. This might be considered one of the limitations of the study, and further research might evaluate the impact of different packaging conditions on the product's shelf life.

In line with the instrumental results, restructured salmon containing CSS (both industrial and artisanal) presented significantly higher color scores throughout storage (*p* < 0.05), reflecting a darker appearance than the control. This outcome was anticipated and attributed to the presence of melanoidins formed during coffee roasting (Chemat et al. 2024). As recently observed by Özhamamcı and Sayın (2025) and Mesárošová et al. (2025), the incorporation of CSS inherently imparts a brown hue that can influence the visual perception of light‐colored meat matrices. Despite this difference in color, trained‐panel liking did not differ significantly between treatments at Days 1 and 14.

Regarding fish odor, R‐ICS and R‐ACS consistently exhibited significantly lower scores (*p* < 0.05) compared with the control (R‐C) across all storage periods. The reduction in fish odor in silverskin‐treated samples is particularly relevant when correlated with lipid oxidation data, as oxidative processes are a major source of volatile compounds responsible for undesirable “fishy” and rancid notes (Dai et al. 2024). Thus, the lower fish odor scores are consistent with the lower abundance of volatile aldehydes (such as hexanal and heptanal) previously described.

Perception of rancid flavor peaked in the control on Day 14 (2.08), whereas treated samples showed lower values, namely, 1.18 for R‐ICS and 0.74 for R‐ACS, both significantly lower than R‐C (*p* < 0.05). WOF, however, the response varied with storage time. On Day 7, R‐ACS showed a significantly higher score (4.44 ± 1.83) than R‐C (3.03 ± 1.38) and R‐ICS (2.31 ± 1.67). Such temporal variation may reflect the dynamic nature of sensory responses associated with lipid oxidation during storage (Byrne et al. 2002; Yan et al. 2026). By Day 14, WOF was more pronounced in the control (3.59), whereas R‐ACS (2.07) and R‐ICS (2.11) showed lower scores. Although R‐ACS showed a higher WOF score on Day 7, this difference was not maintained at the end of storage, when both CSS‐containing formulations showed lower WOF scores than the control.

Other sensory attributes revealed less favorable changes associated with CSS incorporation. Bitterness was significantly higher in R‐ICS and R‐ACS than in R‐C on Days 1 and 7 (*p* < 0.05), whereas on Day 14, only R‐ACS remained significantly higher than R‐C. Similarly, both CSS‐containing treatments showed significantly lower fresh‐fish flavor scores than the control throughout storage (*p* < 0.05), with R‐ACS showing a further reduction compared with R‐ICS on Days 1 and 14. These findings indicate that CSS incorporation modified bitterness and fresh‐fish flavor in the restructured salmon.

Although fish is predominantly consumed fresh, the availability of convenient fish‐based products remains limited. Coupled with challenges such as lipid oxidation and sensory deterioration during storage, this highlights the need for technological strategies that ensure product quality and delay oxidative spoilage (Gautam et al. 2025).

Furthermore, as detailed in Table 3, basic taste attributes, such as saltiness and sourness, did not exhibit significant differences (*p* > 0.05), indicating that the incorporation of CSS did not significantly affect these fundamental taste attributes of the restructured salmon products. Concerning trained‐panel liking, no significant differences among treatments were observed at Days 1 and 14. However, the control exhibited a significant decline by Day 14 (from 6.83 to 5.54), whereas no significant changes across storage were observed for the silverskin formulations (R‐ACS: 5.88–5.40; R‐ICS: 5.89–5.68). Nevertheless, the higher bitterness and lower fresh‐fish flavor observed in CSS‐containing samples indicate that CSS incorporation modified some sensory characteristics, although these changes did not result in lower trained‐panel liking at the evaluated storage times.

**TABLE 3:** Sensory analysis of control, artisanal, and industrial treatments stored at 3°C for 14 days.

	Days								
	1			7			14		
Attributes	R‐C	R‐ICS	R‐ACS	R‐C	R‐ICS	R‐ACS	R‐C	R‐ICS	R‐ACS
Color	1.78 ± 0.86^b.x^	7.00 ± 1.81^a.x^	7.05 ± 1.60^a.x^	1.34 ± 0.41^c.x^	7.10 ± 1.45^b.x^	8.12 ± 0.87^a.x^	1.57 ± 0.63^b.x^	6.94 ± 1.59^a.x^	7.41 ± 1.77^a.x^
Fish odor	8.43 ± 0.89^a.x^	5.22 ± 2.21^b.x^	4.63 ± 2.08^b.x^	8.11 ± 1.75^a.xy^	5.46 ± 1.80^b.x^	4.02 ± 2.97^b.x^	7.14 ± 1.58^a.y^	4.35 ± 1.31^b.x^	3.31 ± 1.45^b.x^
Juiciness	6.77 ± 1.48^a.x^	5.39 ± 2.13^ab.x^	5.30 ± 1.74^b.x^	5.98 ± 2.39^a.x^	5.37 ± 1.28^a.x^	5.37 ± 1.28^a.x^	6.19 ± 1.99^a.x^	5.99 ± 1.67^a.x^	5.37 ± 1.73^a.x^
Fibrousness	3.22 ± 1.65^a.x^	4.17 ± 2.11^a.x^	3.31 ± 1.84^a.x^	4.19 ± 2.13^a.x^	3.81 ± 1.88^a.x^	3.98 ± 2.48^a.x^	3.85 ± 0.92^a.x^	2.84 ± 0.89^b.x^	4.26 ± 1.40^a.x^
Fresh fish flavor	7.31 ± 1.44^a.x^	4.87 ± 1.86^b.x^	3.21 ± 1.69^c.y^	7.88 ± 1.03^a.x^	5.63 ± 1.72^b.x^	4.57 ± 1.83^b.x^	6.03 ± 1.50^a.y^	4.59 ± 1.42^b.x^	2.66 ± 1.13^c.y^
Rancid flavor	0.63 ± 0.36^a.y^	0.62 ± 0.49^a.y^	1.04 ± 1.00^a.y^	2.04 ± 1.21^a.x^	1.53 ± 0.94^a.x^	2.01 ± 1.36^a.x^	2.08 ± 1.18^a.x^	1.18 ± 0.93^b.xy^	0.74 ± 0.57^b.y^
WOF	1.91 ± 1.30^a.y^	2.11 ± 1.44^a.x^	2.61 ± 1.72^a.y^	3.03 ± 1.38^b.x^	2.31 ± 1.67^b.x^	4.44 ± 1.83^a.x^	3.59 ± 1.13^a.x^	2.11 ± 0.82^b.x^	2.07 ± 1.00^b.y^
Saltiness	4.77 ± 2.08^a.x^	4.52 ± 1.94^a.xy^	4.18 ± 1.75^a.x^	4.83 ± 1.98^a.x^	4.81 ± 1.64^a.x^	4.51 ± 1.68^a.x^	4.56 ± 1.73^a.x^	3.39 ± 1.29^a.y^	3.90 ± 1.16^a.x^
Sourness	0.58 ± 0.51^a.x^	0.45 ± 0.27^a.y^	0.63 ± 0.53^a.y^	0.87 ± 0.46^a.x^	0.53 ± 0.44^a.y^	0.74 ± 0.52^a.xy^	0.96 ± 0.89^a.x^	1.18 ± 0.67^a.x^	1.25 ± 1.05^a.x^
Bitterness	0.45 ± 0.50^b.y^	1.31 ± 1.17^a.x^	1.32 ± 1.28^a.x^	0.30 ± 0.24^b.y^	1.67 ± 1.23^a.x^	1.08 ± 0.80^a.y^	1.10 ± 0.88^b.x^	1.64 ± 1.54^ab.x^	2.64 ± 1.53^a.x^
Liking	6.83 ± 1.36^a.x^	5.89 ± 1.41^a.x^	5.88 ± 1.61^a.x^	6.79 ± 1.35^a.x^	5.36 ± 1.44^b.x^	5.79 ± 1.66^ab.x^	5.54 ± 1.07^a.y^	5.68 ± 1.64^a.x^	5.40 ± 0.88^a.x^

*Note*: Different letters (^a,b,c^) show statistically differences between treatments within each storage time; different letters (^x,y,z^) indicate statistically differences between storage time within each treatment.

Abbreviation: WOF, warmed‐over flavor.

As an exploratory visualization, the principal component analysis (PCA) (Figure 4), which accounted for 70.87% of the total variance, suggested associations between silverskin‐treated samples and distinct sensory attributes during storage. The artisanal treatment (R‐ACS) was positioned in the PCA in association with more intense color and reduced rancidity, suggesting an association with lower perceived rancidity. Conversely, the industrial treatment (R‐ICS) showed associations with bitterness and fibrousness. The CSS‐containing treatments were also associated with higher bitterness and lower fresh‐fish flavor. The control sample (R‐C) was primarily linked to fresh fish odor and flavor but also presented higher levels of rancidity and warmed‐over flavor at the end of storage, which was consistent with the oxidative trends observed in the TBARS and volatile compound analyses.

**FIGURE 4:**
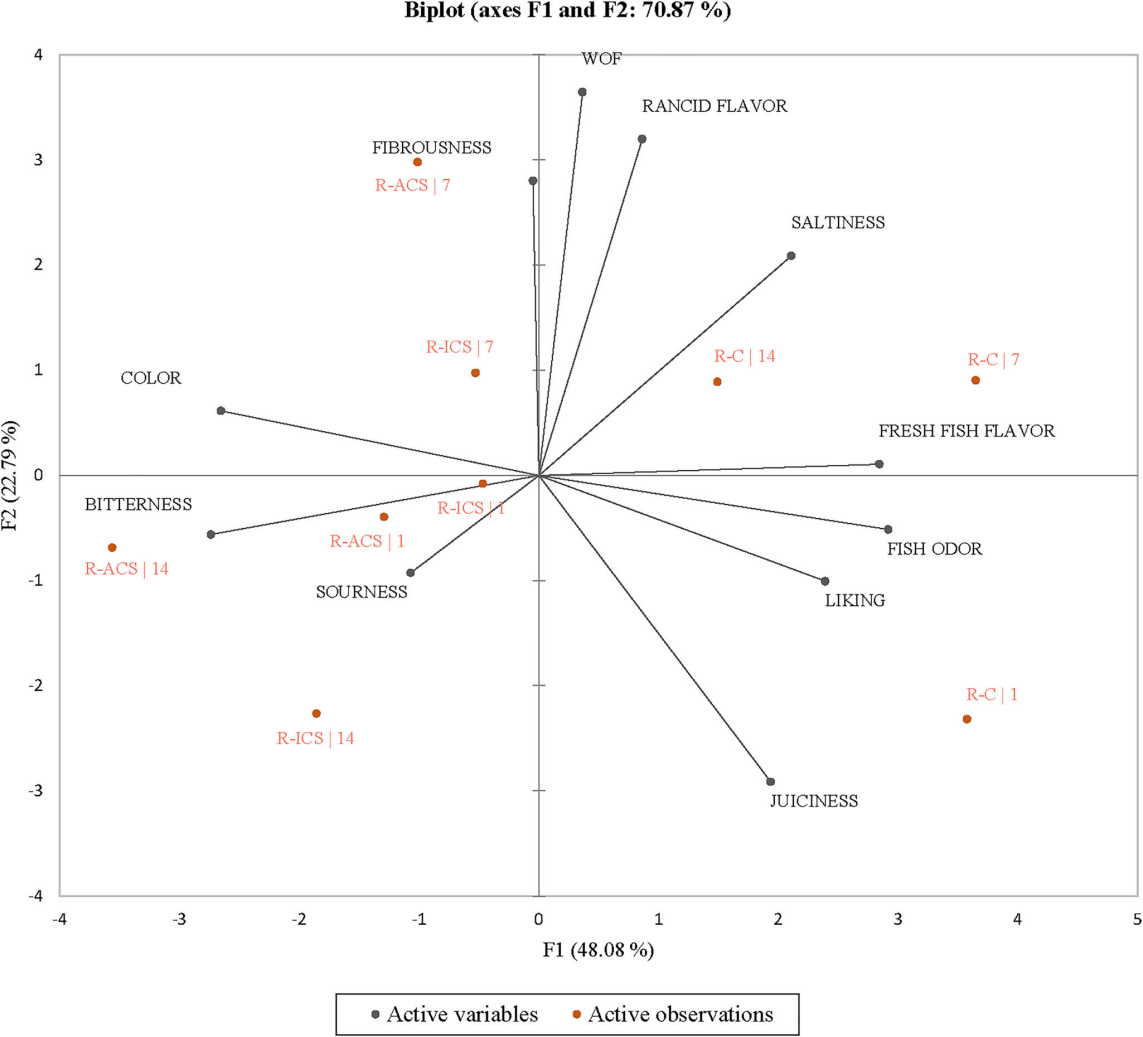
Principal component analysis (PCA) biplot of sensory characteristics (Factors F1 and F2) of restructured salmon products at different storage times. R‐C = Control restructured salmon; R‐ICS = Restructured salmon with industrial coffee silverskin; R‐ACS = Restructured salmon with artisanal coffee silverskin. WOF, warmed‐over flavor.

## 4 Conclusion

This study supports the potential of CSS as a sustainable source of antioxidant compounds for improving the oxidative stability of restructured salmon. The two CSS samples exhibited distinct antioxidant‐related profiles, with ACS showing higher TPC, DPPH, and ABTS values, whereas ICS exhibited higher FRAP. These differences are specific to the evaluated samples and cannot be attributed solely to roasting conditions. Under the experimental conditions, both CSS sources were associated with lower lipid oxidation in restructured salmon during refrigerated storage. Sensory responses varied across storage time, whereas CSS incorporation increased bitterness and reduced fresh‐fish flavor. The DNPH results did not allow a definitive conclusion regarding the effect of CSS on protein oxidation. The use of a single concentration represents a limitation, as it does not allow dose optimization or identification of potential prooxidant thresholds. The microbiological results supported the sensory evaluation period of up to 14 days under the conditions studied. Further studies should evaluate multiple CSS concentrations and combined preservation strategies (hurdle technologies) to optimize sensory acceptance and support broader application in fish products.

## Author Contributions

**Antonia Dayane Jenyffer de Farias Marques**: conceptualization, investigation, methodology, formal analysis, writing – original draft, data curation. **Mario Estevez**: conceptualization, funding acquisition, writing – review and editing, project administration, resources, supervision. **José María Martín‐miguélez**: formal analysis. **Andrea Sánchez‐Canales**: formal analysis. **Eike Guilherme Torres de Souza**: formal analysis, software. **Sonia Ventanas**: data curation. **Josué Delgado**: data curation. **Mercia de Sousa Galvão**: data curation. **Marcos dos Santos Lima**: formal analysis. **Taliana Kênia Alencar Bezerra**: conceptualization, funding acquisition, writing – review and editing, project administration, resources, supervision. **David Morcuende**: conceptualization, investigation, writing – review and editing, project administration, supervision.

## Conflicts of Interest

The authors declare no conflicts of interest.

## Supporting information



Supplementary Information: jfds71577‐sup‐0001‐SuppMat.docx

## References

[jfds71577-bib-0001] AOAC International . 2023. Official Methods of Analysis 22nd Edition 2023. AOAC International.

[jfds71577-bib-0002] Abreu, T. L. , M. Estévez , L. M. Carvalho , et al. 2024. “Unveiling the Bioactivity and Bioaccessibility of Phenolic Compounds From Organic Coffee Husks Using an in Vitro Digestion Model.” Journal of the Science of Food and Agriculture 104, no. 3: 1833–1842. 10.1002/jsfa.13078. 37884474

[jfds71577-bib-0003] Alcantara, G. M. R. N. , L. C. Martins , W. P. C. Gomes , D. Dresch , F. R. P. Rocha , and W. R. Melchert . 2025. “Effect of Roasting on Chemical Composition of Coffee.” Food Chemistry 477, no. (June): 143169. 10.1016/j.foodchem.2025.143169. 40010185

[jfds71577-bib-0004] Alhammad, K. , N. Howell , and N. Haq . 2021. “The Effect of ‘Green’ ANTIOXIDANTS on Lipid Oxidation in Dried Salmon (*Salmo salar*) During Storage Conditions.” Journal of Experimental Biology and Agricultural Sciences 9, no. 2: 206–213. 10.18006/2021.9(2).206.213.

[jfds71577-bib-0005] Ateş, G. , and Y. Elmacı . 2019. “Physical, Chemical and Sensory Characteristics of Fiber‐Enriched Cakes Prepared With Coffee Silverskin as Wheat Flour Substitution.” Journal of Food Measurement and Characterization 13, no. 1: 755–763. 10.1007/s11694-018-9988-9.

[jfds71577-bib-0006] Benzie, I. F. , and J. J. Strain . 1999. “Ferric Reducing/Antioxidant Power Assay: Direct Measure of Total Antioxidant Activity of Biological Fluids and Modified Version for Simultaneous Measurement of Total Antioxidant Power and Ascorbic Acid Concentration.” Methods in Enzymology 299: 15–27. 10.1016/s0076-6879(99)99005-5. 9916193

[jfds71577-bib-0007] Billmeyer Jr., F. W. 1988. “Quantifying Color Appearance Visually and Instrumentally.” Color Research & Application 13, no. 3: 140–145. 10.1002/col.5080130306.

[jfds71577-bib-0008] Bolchini, S. , L. Angeli , G. Ferrentino , et al. 2025. “Free Radical Scavenging Kinetics of Maillard Reaction Products: A Glucose‐Glycine Model System.” LWT 217, no. (February): 117316. 10.1016/j.lwt.2024.117316.

[jfds71577-bib-0009] Bondam, A. F. , D. D. Silveira , J. P. Santos , and J. F. Hoffmann . 2022. “Phenolic Compounds From Coffee By‐Products: Extraction and Application in the Food and Pharmaceutical Industries.” Trends in Food Science & Technology 123, no. (May): 172–186. 10.1016/j.tifs.2022.03.013.

[jfds71577-bib-0010] Bourne, M. C. 1978. “Texture Profile Analysis.” Food Technology 32: 62–66.

[jfds71577-bib-0011] Brand‐Williams, W. , M. E. Cuvelier , and C. Berset . 1995. “Use of a Free Radical Method to Evaluate Antioxidant Activity.” LWT—Food Science and Technology 28, no. 1: 25–30. 10.1016/S0023-6438(95)80008-5.

[jfds71577-bib-0012] Byrne, D. V. , W. L. P. Bredie , D. S. Mottram , and M. Martens . 2002. “Sensory and Chemical Investigations on the Effect of Oven Cooking on Warmed‐Over Flavour Development in Chicken Meat.” Meat Science 61, no. 2: 127–139. 10.1016/S0309-1740(01)00171-1. 22064001

[jfds71577-bib-0013] Cao, Q. , H. Du , Y. Huang , et al. 2019. “The Inhibitory Effect of Chlorogenic Acid on Lipid Oxidation of Grass Carp (*Ctenopharyngodon idellus*) During Chilled Storage.” Food and Bioprocess Technology 12: 2050–2061. 10.1007/s11947-019-02365-0.

[jfds71577-bib-0014] Chemat, A. , M. Schweiger , D. Touraud , et al. 2024. “Cascade Extractions of Coffee Silverskin: Towards Zero Solid Waste Valorization of a Byproduct.” Sustainable Chemistry and Pharmacy 42, no. (December): 101779. 10.1016/j.scp.2024.101779.

[jfds71577-bib-0015] Chen, C. , Y. Wu , J. Li , et al. 2023. “TBtools‐II: A ‘One for All, All for One’ Bioinformatics Platform for Biological Big‐Data Mining.” Molecular Plant 16, no. 11: 1733–1742. 10.1016/j.molp.2023.09.010. 37740491

[jfds71577-bib-0016] Cilli, L. P. , L. R. F. Contini , P. Sinnecker , et al. 2020. “Effects of Grape Pomace Flour on Quality Parameters of Salmon Burger.” Journal of Food Processing and Preservation 44, no. 2: e14329. 10.1111/jfpp.14329.

[jfds71577-bib-0017] Cirne, C. T. , M. H. Tunick , and R. E. Trout . 2019. “The Chemical and Attitudinal Differences Between Commercial and Artisanal Products.” npj Science of Food 3, no. 1: 19. 10.1038/s41538-019-0053-9. PMC6731266 31508494

[jfds71577-bib-0018] Costa, A. S. G. , R. C. Alves , A. F. Vinha , et al. 2018. “Nutritional, Chemical and Antioxidant/Pro‐Oxidant Profiles of Silverskin, a Coffee Roasting By‐Product.” Food Chemistry 267, no. (November): 28–35. 10.1016/j.foodchem.2017.03.106. 29934169

[jfds71577-bib-0019] Dai, W. , S. He , L. Huang , et al. 2024. “Strategies to Reduce Fishy Odor in Aquatic Products: Focusing on Formation Mechanism and Mitigation Means.” Food Chemistry 444, no. (June): 138625. 10.1016/j.foodchem.2024.138625. 38325089

[jfds71577-bib-0020] Dudonné, S. , X. Vitrac , P. Coutière , M. Woillez , and J.‐M. Mérillon . 2009. “Comparative Study of Antioxidant Properties and Total Phenolic Content of 30 Plant Extracts of Industrial Interest Using DPPH, ABTS, FRAP, SOD, and ORAC Assays.” Journal of Agricultural and Food Chemistry 57, no. 5: 1768–1774. 10.1021/jf803011r. 19199445

[jfds71577-bib-0021] Estévez, M. 2011. “Protein Carbonyls in Meat Systems: A Review.” Meat Science 89, no. 3: 259–279. 10.1016/j.meatsci.2011.04.025. 21621336

[jfds71577-bib-0022] Estévez, M. 2021. “Critical Overview of the Use of Plant Antioxidants in the Meat Industry: Opportunities, Innovative Applications and Future Perspectives.” Meat Science 181, no. (November): 108610. 10.1016/j.meatsci.2021.108610. 34147961

[jfds71577-bib-0023] Estévez, M. , S. Ventanas , and R. Cava . 2005. “Protein Oxidation in Frankfurters With Increasing Levels of Added Rosemary Essential Oil: Effect on Color and Texture Deterioration.” Journal of Food Science 70, no. 7: c427–c432. 10.1111/j.1365-2621.2005.tb11464.x.

[jfds71577-bib-0039] Farias Marques, A. D. J. , J. L. Tavares , L. M. Carvalho , et al. 2022. “Oxidative Stability of Chicken Burgers Using Organic Coffee Husk Extract.” Food Chemistry 393, no. (November): 133451. 10.1016/j.foodchem.2022.133451. 35751207

[jfds71577-bib-0025] Folch, J. , M. Lees , and G. H. Sloane Stanley . 1957. “A Simple Method for the Isolation and Purification of Total Lipides From Animal Tissues.” Journal of Biological Chemistry 226, no. 1: 497–509. 10.1016/S0021-9258(18)64849-5. 13428781

[jfds71577-bib-0026] Food and Agriculture Organization (FAO) . 2024. Food Waste Index Report 2024 . Food and Agriculture Organization. https://www.fao.org/family‐farming/detail/en/c/1681058/.

[jfds71577-bib-0027] Ganhão, R. , M. Estévez , and D. Morcuende . 2011. “Suitability of the TBA Method for Assessing Lipid Oxidation in a Meat System With Added Phenolic‐Rich Materials.” Food Chemistry 126, no. 2: 772–778. 10.1016/j.foodchem.2010.11.064.

[jfds71577-bib-0028] Gautam, R. K. , A. S. Kakatkar , A. Debbarma , et al. 2025. “Shelf‐Life Extension of Ready‐to‐Cook Tilapia (*Oreochromis* spp.) Fish Balls Using Hurdle Technology.” Food Bioscience 63, no. (January): 105739. 10.1016/j.fbio.2024.105739.

[jfds71577-bib-0029] Gemechu, F. G. 2020. “Embracing Nutritional Qualities, Biological Activities and Technological Properties of Coffee Byproducts in Functional Food Formulation.” Trends in Food Science & Technology 104, no. (October): 235–261. 10.1016/j.tifs.2020.08.005.

[jfds71577-bib-0030] Gouvêa, F. J. , V. S. Oliveira , B. J. Mariano , et al. 2023. “Natural Antioxidants as Strategy to Minimize the Presence of Lipid Oxidation Products in Canned Fish: Research Progress, Current Trends and Future Perspectives.” Food Research International 173, no. (November): 113314. 10.1016/j.foodres.2023.113314. 37803625

[jfds71577-bib-0031] Grünenwald, M. , M. B. Adams , C. G. Carter , et al. 2019. “Pigment‐Depletion in Atlantic Salmon (*Salmo salar*) Post‐Smolt Starved at Elevated Temperature Is Not Influenced by Dietary Carotenoid Type and Increasing α‐Tocopherol Level.” Food Chemistry 299, no. (November): 125140. 10.1016/j.foodchem.2019.125140. 31299520

[jfds71577-bib-0032] Günal‐Köroğlu, D. , H. Yılmaz , B. G. Subasi , and E. Capanoglu . 2025. “Protein Oxidation: The Effect of Different Preservation Methods or Phenolic Additives During Chilled and Frozen Storage of Meat/Meat Products.” Food Research International 200, no. (January): 115378. 10.1016/j.foodres.2024.115378. 39779159

[jfds71577-bib-0033] Haq, M. , M. Sadek Ali , J.‐S. Park , J.‐W. Kim , W. Zhang , and B.‐S. Chun . 2025. “Atlantic Salmon (*Salmo salar*) Waste as a Unique Source of Biofunctional Protein Hydrolysates: Emerging Productions, Promising Applications, and Challenges Mitigation.” Food Chemistry 462, no. (January): 141017. 10.1016/j.foodchem.2024.141017. 39216379

[jfds71577-bib-0034] Indio, V. , F. Savini , F. Gardini , et al. 2024. “Microbiological Safety of Dry‐Cured Fish From the Raw Material to the End of Processing.” International Journal of Food Microbiology 415, no. (April): 110641. 10.1016/j.ijfoodmicro.2024.110641. 38432054

[jfds71577-bib-0035] Kang, M. J. , M. J. Kim , H. S. Kwak , and S. S. Kim . 2019. “Effects of Milling Methods and Cultivars on Physicochemical Properties of Whole‐Wheat Flour.” Journal of Food Quality 2019, no. 1: 3416905. 10.1155/2019/3416905.

[jfds71577-bib-0036] Kašpar, M. , J. Kubáleková , S. Řezková , T. Bajer , and P. Česla . 2025. “Optimization of Gradient Separation Conditions Using Liquid Chromatography With Tandem Mass Spectrometric Detection for Analysis of Phenolic Profile During Coffee Roasting.” Journal of Chromatography A 1756, no. (August): 466059. 10.1016/j.chroma.2025.466059. 40408785

[jfds71577-bib-0037] Li, Y. , S. Xiao , Q. Zhang , N. Wang , Q. Yang , and J. Hao . 2024. “Development and Standardization of Spectrophotometric Assay for Quantification of Thermal Hydrolysis‐Origin Melanoidins and Its Implication in Antioxidant Activity Evaluation.” Journal of Hazardous Materials 476, no. (September): 135021. 10.1016/j.jhazmat.2024.135021. 38944987

[jfds71577-bib-0038] Mafe, A. N. , G. I. Edo , R. S. Makia , et al. 2024. “A Review on Food Spoilage Mechanisms, Food Borne Diseases and Commercial Aspects of Food Preservation and Processing.” Food Chemistry Advances 5, no. (December): 100852. 10.1016/j.focha.2024.100852.

[jfds71577-bib-0040] Martuscelli, M. , L. Esposito , and D. Mastrocola . 2021. “The Role of Coffee Silver Skin Against Oxidative Phenomena in Newly Formulated Chicken Meat Burgers after Cooking.” Foods 10, no. 8: 1833. 10.3390/foods10081833. PMC8394139 34441610

[jfds71577-bib-0041] Medina, I. , J. M. Gallardo , M. J. González , S. Lois , and N. Hedges . 2007. “Effect of Molecular Structure of Phenolic Families as Hydroxycinnamic Acids and Catechins on Their Antioxidant Effectiveness in Minced Fish Muscle.” Journal of Agricultural and Food Chemistry 55: 3889–3895. 10.1021/jf063498i. 17451250

[jfds71577-bib-0042] Mesárošová, A. , L. Jurčaga , A. Bobková , et al. 2025. “Coffee Silverskin: Nontraditional Natural Antioxidant for the Meat Industry.” Applied Food Research 5, no. 2: 101185. 10.1016/j.afres.2025.101185.

[jfds71577-bib-0043] Montenegro Gallazzi, L. F. , C. D. Pérez , L. Rossetti , S. Cunzolo , S. A. Rizzo , and A. M. Descalzo . 2025. “The Pasture System Improves Natural Antioxidants and Functional Lipids While Reducing Lipid Peroxidation in *Ctenopharyngodon idella* (Grass Carp) Burgers During Storage.” Food Chemistry 464, no. (February): 141866. 10.1016/j.foodchem.2024.141866. 39509892

[jfds71577-bib-0044] Mozzon, M. , R. Foligni , C. Mannozzi , and S. Vittori . 2024. “Assessment of Lipid Oxidation in Fish and Fish Products Processed by Cold Plasma Technologies.” Applied Food Research 4, no. 2: 100646. 10.1016/j.afres.2024.100646.

[jfds71577-bib-0045] Muñoz, A. E. , S. S. Hernández , A. R. Tolosa , S. P. Burillo , and M. O. Herrera . 2020. “Evaluation of Differences in the Antioxidant Capacity and Phenolic Compounds of Green and Roasted Coffee and Their Relationship With Sensory Properties.” LWT 128, no. (June): 109457. 10.1016/j.lwt.2020.109457.

[jfds71577-bib-0046] Nolasco, A. , J. Squillante , S. Velotto , et al. 2022. “Valorization of Coffee Industry Wastes: Comprehensive Physicochemical Characterization of Coffee Silverskin and Multipurpose Recycling Applications.” Journal of Cleaner Production 370, no. (October): 133520. 10.1016/j.jclepro.2022.133520.

[jfds71577-bib-0047] Nzekoue, F. K. , S. Angeloni , L. Navarini , et al. 2020. “Coffee Silverskin Extracts: Quantification of 30 Bioactive Compounds by a New HPLC‐MS/MS Method and Evaluation of Their Antioxidant and Antibacterial Activities.” Food Research International 133, no. (July): 109128. 10.1016/j.foodres.2020.109128. 32466943

[jfds71577-bib-0048] Ortuño, J. , L. Mateo , M. T. Rodríguez‐Estrada , and S. Bañón . 2021. “Effects of *Sous vide* vs. Grilling Methods on Lamb Meat Colour and Lipid Stability During Cooking and Heated Display.” Meat Science 171, no. (January): 108287. 10.1016/j.meatsci.2020.108287. 32896774

[jfds71577-bib-0049] Özhamamcı, İ. , and B. Sayın . 2025. “Circular Utilization of Coffee Silverskin in Beef Patties: Effects on Technological, Sensory, and Safety Attributes.” Food Bioscience 72, no. (October): 107436. 10.1016/j.fbio.2025.107436.

[jfds71577-bib-0050] Padilha, C. V. S. , G. A. Miskinis , M. E. A. O. Souza , et al. 2017. “Rapid Determination of Flavonoids and Phenolic Acids in Grape Juices and Wines by RP‐HPLC/DAD: Method Validation and Characterization of Commercial Products of the New Brazilian Varieties of Grape.” Food Chemistry 228, no. (August): 106–115. 10.1016/j.foodchem.2017.01.137. 28317702

[jfds71577-bib-0051] Pateiro, M. , J. A. Gómez‐Salazar , M. Jaime‐Patlán , M. E. Sosa‐Morales , and J. M. Lorenzo . 2021. “Plant Extracts Obtained With Green Solvents as Natural Antioxidants in Fresh Meat Products.” Antioxidants 10, no. 2: 181. 10.3390/antiox10020181. PMC7912489 33513904

[jfds71577-bib-0052] Qi, J. , Y. Xu , W. Zhang , X. Xie , G. Xiong , and X. Xu . 2021. “Short‐Term Frozen Storage of Raw Chicken Meat Improves Its Flavor Traits Upon Stewing.” LWT 142, no. (May): 111029. 10.1016/j.lwt.2021.111029.

[jfds71577-bib-0053] Re, R. , N. Pellegrini , A. Proteggente , A. Pannala , M. Yang , and C. Rice‐Evans . 1999. “Antioxidant Activity Applying an Improved ABTS Radical Cation Decolorization Assay.” Free Radical Biology and Medicine 26, no. 9: 1231–1237. 10.1016/S0891-5849(98)00315-3. 10381194

[jfds71577-bib-0054] Ren, Z. , Z. Li , Z. Hu , et al. 2024. “Recent Insights Into Bonding Technologies in Restructured Meat Production: A Review.” Food Chemistry: X 23, no. (October): 101712. 10.1016/j.fochx.2024.101712. PMC11363562 39220417

[jfds71577-bib-0024] Ripke Ferreira, R. , B. H. F. Saqueti , P. D. S. Santos , et al. 2022. “Effect of *Salvia (Salvia officinalis)* on the Oxidative Stability of Salmon Hamburgers.” LWT 154, no. (January): 112867. 10.1016/j.lwt.2021.112867.

[jfds71577-bib-0055] Sánchez‐Alonso, I. , M. Careche , P. Moreno , M. Jesús González , and I. Medina . 2011. “Testing Caffeic Acid as a Natural Antioxidant in Functional Fish‐Fibre Restructured Products.” LWT—Food Science and Technology 44, no. 4: 1149–1155. 10.1016/j.lwt.2010.11.018.

[jfds71577-bib-0056] Sandler, S. R. , and W. Karo . 1992. Sourcebook of Advanced Organic Laboratory Preparations. Academic Press.

[jfds71577-bib-0057] Silva, M. O. , J. N. B. Honfoga , L. L. de Medeiros , M. S. Madruga , and T. K. A. Bezerra . 2020. “Obtaining Bioactive Compounds From the Coffee Husk (*Coffea arabica* L.) Using Different Extraction Methods.” Molecules 26, no. 1: 46. 10.3390/molecules26010046. PMC7795416 33374108

[jfds71577-bib-0058] Thorsen, M. , M. Mirosa , S. Skeaff , F. Goodman‐Smith , and P. Bremer . 2024. “Upcycled Food: How Does It Support the Three Pillars of Sustainability?” Trends in Food Science & Technology 143, no. (January): 104269. 10.1016/j.tifs.2023.104269.

[jfds71577-bib-0059] Tieghi, H. , L. A. Pereira , G. S. Viana , et al. 2024. “Effects of Geographical Origin and Post‐Harvesting Processing on the Bioactive Compounds and Sensory Quality of Brazilian Specialty Coffee Beans.” Food Research International 186, no. (June): 114346. 10.1016/j.foodres.2024.114346. 38729720

[jfds71577-bib-0060] Tokay, F. G. , A. C. Alp , and P. Yerlikaya . 2021. “Production and Shelf Life of Restructured Fish Meat Binded by Microbial Transglutaminase.” LWT 152, no. (December): 112369. 10.1016/j.lwt.2021.112369.

[jfds71577-bib-0061] Vargas‐Sánchez, R. D. , B. M. Torres‐Martínez , G. Ramón , and G. R. Torrescano‐Urrutia , 2024. “Utilization of Coffee Silverskin Aqueous Extract to Improve Pork Meat Homogenates Oxidative Stability.” Biotecnia 26, no. (December): 42–49. 10.18633/biotecnia.v26i1.2128.

[jfds71577-bib-0062] Vignoli, J. A. , D. G. Bassoli , and M. T. Benassi . 2011. “Antioxidant Activity, Polyphenols, Caffeine and Melanoidins in Soluble Coffee: The Influence of Processing Conditions and Raw Material.” Food Chemistry 124, no. 3: 863–868. 10.1016/j.foodchem.2010.07.008.

[jfds71577-bib-0063] Wu, H. , Z. Liu , P. Lu , C. Barrow , F. R. Dunshea , and H. A. R. Suleria . 2022. “Bioaccessibility and Bioactivities of Phenolic Compounds From Roasted Coffee Beans During In Vitro Digestion and Colonic Fermentation.” Food Chemistry 386, no. (August): 132794. 10.1016/j.foodchem.2022.132794. 35349898

[jfds71577-bib-0064] Yan, X. , Z. Ma , Y. Zhu , et al. 2026. “Impact of Protein Succinylation and Myofibrillar Protein‐Lipid Co‐Oxidation Interactions on Heterocyclic Amine Formation of Thermally Processed Goose Meat.” Food Research International 242, no. (October): 120260. 10.1016/j.foodres.2026.120260. 42637388

[jfds71577-bib-0065] Zhang, K. , N. Li , Z. Wang , et al. 2024. “Recent Advances in the Color of Aquatic Products: Evaluation Methods, Discoloration Mechanism, and Protection Technologies.” Food Chemistry 434, no. (February): 137495. 10.1016/j.foodchem.2023.137495. 37741243

[jfds71577-bib-0066] Zoccatelli, G. , and M. Ciulu . 2025. “Perspectives on the Use of Coffee Silverskin in Food Formulations.” Journal of Agriculture and Food Research 21, no. (June): 101975. 10.1016/j.jafr.2025.101975.

