# Supplementary material for: Coffee (Coffea arabica L.) Silverskin: Transforming a Recycled By‐Product Into Natural Antioxidant Ingredients for Ready‐to‐Eat Fish Products
Source: J Food Sci. 2026 Oct 21;91(10):e71577. doi: 10.1111/1750-3841.71577 (PMC13686113; doi:10.1111/1750-3841.71577)
Supplement: Supplementary file 1 [file JFDS-91-0-s001.docx]

Supplementary Table 1. Relative particle size distribution (%) of milled artisanal (ACS) and industrial (ICS) coffee silverskin obtained by laser diffraction analysis.

| **Particle Size (μm)** | **ICS (%)** | **ACS (%)** | **Overall Average (%)** |
| --- | --- | --- | --- |
| 0.06 | 8.81±0.35 | 7.36±0.49 | 8.08±0.90 |
| 3.9 | 7.32±0.04 | 4.74±0.31 | 6.03±1.38 |
| 7.8 | 10.14±0.06 | 8.21±0.58 | 9.17±1.07 |
| 15.6 | 17.22±0.16 | 18.27±1.16 | 17.75±0.96 |
| 31.0 | 23.12±0.06 | 25.91±1.77 | 24.52±1.83 |
| 63.0 | 18.85±0.26 | 20.19±0.87 | 19.52±0.95 |
| 125.0 | 13.54±0.47 | 14.02±2.75 | 13.78±1.81 |
| 250.0 | 0.99±0.01 | 1.30±0.86 | 1.15±0.62 |
| Mean Particle Size (*μ*m) | 42.23±0.51 | 45.20±5.19 | 43.71±3.68 |
| Fraction 7.8 – 125 *μ*m (%) | 82.88±0.38 | 86.59±0.07 | 84.74±2.05 |

**Note(s):** ICS = Industrial coffee silverskin; ACS = Artisanal coffee silverskin. Results are expressed as mean ± standard deviation (n = 3).

Supplementary Table 2. Volatile compounds obtained (by area) from restructured salmon products.

| Compounds | CAS | IRL | R-C | | | | | | | | | R-ICS | | | | | | | | | R-ACS | | | | | | | | *p-*value | | | | | |
| --- | --- | --- | --- | --- | --- | --- | --- | --- | --- | --- | --- | --- | --- | --- | --- | --- | --- | --- | --- | --- | --- | --- | --- | --- | --- | --- | --- | --- | --- | --- | --- | --- | --- | --- |
|  |  |  | T1 | T14 | | | T28 | | | T1 | | | T14 | | | T28 | | | T1 | | | T14 | | | T28 | | | C | | T | | C*T | | |
| Acetaldehyde | 75-07-0 | 404 | 7.57E+04 ab | 9.62E+04 a | | | 6.64E+04 ab | | | 8.09E+04 a | | | 8.09E+04 a | | | 6.64E+04 ab | | | 4.42E+04 bc | | | 7.56E+04 ab | | | 2.67E+04 bc | | | **< 0.0001** | | **< 0.0001** | | 0.123 | | |
| Propanal | 123-38-6 | 461 | 1.86E+05 c | 2.94E+05 a | | | 2.10E+05 b | | | n.d | | | n.d | | | n.d | | | n.d | | | n.d | | | n.d | | | **< 0.0001** | | **< 0.0001** | | **< 0.0001** | | |
| Butanal | 123-72-8 | 593 | 1.46E+04 b | 3.25E+04 a | | | 3.27E+04 a | | | n.d | | | n.d | | | n.d | | | n.d | | | n.d | | | n.d | | | **< 0.0001** | | **< 0.0001** | | **< 0.0001** | | |
| 2-Butanone | 78-93-3 | 598 | 1.93E+04 de | 3.69E+04 b | | | 3.56E+04 bc | | | 5.88E+04 a | | | 5.27E+04 a | | | 6.47E+04 a | | | 1.30E+04 e | | | 2.22E+04 cde | | | 3.17E+04 bcd | | | **< 0.0001** | | **< 0.0001** | | **0.006** | | |
| 2-Methylfuran | 534-22-5 | 606 | 2.64E+03 b | 3.46E+03 a | | | 1.31E+03 c | | | n.d | | | n.d | | | n.d | | | n.d | | | n.d | | | n.d | | | **< 0.0001** | | **< 0.0001** | | **< 0.0001** | | |
| Ethyl Acetate | 141-78-6 | 629 | n.d | n.d | | | n.d | | | n.d | | | n.d | | | n.d | | | n.d | | | n.d | | | 3.89E+05 a | | | **< 0.0001** | | **< 0.0001** | | **< 0.0001** | | |
| 3-Methylbutanal | 590-86-3 | 652 | 4.76E+04 b | 3.67E+04 c | | | 3.52E+04 c | | | 8.24E+04 a | | | 8.24E+04 a | | | 7.73E+04 a | | | 8.38E+04 a | | | 7.79E+03 d | | | 4.71E+04 b | | | **< 0.0001** | | **< 0.0001** | | **< 0.0001** | | |
| 2-Methylbutanal | 96-17-3 | 662 | 3.19E+04 c | 2.08E+04 de | | | 1.84E+04 e | | | 4.23E+04 b | | | 4.23E+04 b | | | 3.36E+04 c | | | 5.86E+04 a | | | 7.75E+03 f | | | 2.36E+04 d | | | **< 0.0001** | | **< 0.0001** | | **< 0.0001** | | |
| 1-Penten-3-ol | 616-25-1 | 684 | 2.87E+05 c | 5.98E+05 b | | | 7.63E+05 a | | | n.d | | | n.d | | | n.d | | | n.d | | | n.d | | | n.d | | | **< 0.0001** | | **< 0.0001** | | **< 0.0001** | | |
| 2,3-Pentanedione | 600-14-6 | 698 | 1.42E+05 a | 2.29E+05 a | | | 2.38E+05 a | | | n.d | | | n.d | | | n.d | | | n.d | | | n.d | | | n.d | | | **< 0.0001** | | 0.227 | | 0.214 | | |
| Pentanal | 110-62-3 | 700 | 2.80E+05 a | | 3.68E+05 a | | | 3.35E+05 a | | | n.d | | | n.d | | | n.d | | | 7.70E+03 b | | | 3.42E+04 b | | | 7.01E+03 b | | | **< 0.0001** | | 0.117 | | 0.329 | |
| 2-Ethylfuran | 3208-16-0 | 703 | 2.72E+04 b | | 3.52E+04 a | | | 2.88E+04 b | | | n.d | | | n.d | | | n.d | | | n.d | | | n.d | | | n.d | | | **< 0.0001** | | **0.001** | | **< 0.0001** | |
| Propyl acetate | 109-60-4 | 708 | 1.21E+06 b | | n.d | | | n.d | | | n.d | | | n.d | | | n.d | | | 3.73E+06 a | | | n.d | | | n.d | | | **< 0.0001** | | **< 0.0001** | | **< 0.0001** |  |
| 2-Hexanone | 591-78-6 | 790 | 1.60E+05 a | | 7.32E+03 b | | | 1.22E+04 b | | | 9.72E+03 b | | | 8.70E+03 b | | | n.d | | | n.d | | | n.d | | | n.d | | | **< 0.0001** | | **< 0.0001** | | **< 0.0001** |  |
| Hexanal | 66-25-1 | 800 | 2.46E+06 a | | 7.08E+04 b | | | 7.44E+04 b | | | 1.04E+05 b | | | 1.14E+05 b | | | 1.04E+05 b | | | 5.09E+04 b | | | 4.73E+04 b | | | 6.61E+04 b | | | **< 0.0001** | | **< 0.0001** | | **< 0.0001** |  |
| 2-Heptanone | 110-43-0 | 848 | 9.93E+03 c | | 3.52E+04 b | | | 4.21E+04 a | | | n.d | | | n.d | | | n.d | | | n.d | | | n.d | | | n.d | | | **< 0.0001** | | **< 0.0001** | | **< 0.0001** |  |
| (Z)-4-Heptenal | 6728-31-0 | 900 | 2.37E+04 c | | 8.73E+04 b | | | 1.04E+05 a | | | n.d | | | n.d | | | n.d | | | n.d | | | n.d | | | n.d | | | **< 0.0001** | | **< 0.0001** | | **< 0.0001** |  |
| Heptanal | 111-71-7 | 901 | 1.37E+05 c | | 3.83E+05 b | | | 4.11E+05 a | | | 1.77E+04 e | | | 1.77E+04 e | | | 1.77E+04 e | | | 1.40E+04 e | | | 1.82E+04 e | | | 3.28E+04 d | | | **< 0.0001** | | **< 0.0001** | | **< 0.0001** |  |
| Benzaldehyde | 100-52-7 | 962 | 9.03E+03 cd | | 1.48E+04 bcd | | | 2.33E+04 bc | | | 2.62E+04 b | | | 4.41E+04 a | | | 4.41E+04 a | | | n.d | | | n.d | | | n.d | | | **< 0.0001** | | **0.001** | | **0.031** |  |
| 1-Octen-3-ol | 3391-86-4 | 980 | 1.78E+04 c | | 1.97E+05 a | | | 1.59E+05 b | | | n.d | | | n.d | | | n.d | | | n.d | | | 1.93E+04 c | | | 1.46E+04 c | | | **< 0.0001** | | **< 0.0001** | | **< 0.0001** |  |
| Linalool oxide | 5989-33-3 | 1074 | n.d | | n.d | | | n.d | | | 2.46E+04 a | | | 2.67E+04 a | | | 2.46E+04 a | | | n.d | | | n.d | | | n.d | | | **< 0.0001** | | 0.617 | | 0.738 |  |
| Nonanal | 124-19-6 | 1104 | 4.66E+04 b | | 1.66E+05 a | | | 1.63E+05 a | | | 2.14E+04 c | | | 2.14E+04 c | | | 1.93E+04 c | | | 2.19E+04 c | | | 1.32E+04 c | | | 3.18E+04 bc | | | **< 0.0001** | | **< 0.0001** | | **< 0.0001** |  |
|  |  |  |  | | |  | | |  | | |  | | |  | | |  | | |  | | |  | | |  | |  | |  | |  | |

Note(s): R-C = Control restructured salmon (no antioxidants); R-ICS = Restructured salmon with industrial coffee silverskin; R-ACS = Restructured salmon with artisanal coffee silverskin; n.d = not detected. Different lowercase letters within the same row indicate significant differences (p < 0.05) between samples. C = composition; T = time; C*T = composition x time.

Supplementary Table 3. Microbiological counts (log CFU/g) of restructured salmon products during refrigerated storage at 3ºC.

|  |  | Day 1 | Day 7 | Day 14 | Day 21 |
| --- | --- | --- | --- | --- | --- |
| Aerobic mesophilic | R-C | 2.00±0.10 | 2.53±0.56 | 2.18±0.42 | 2.96±0.90 |
|  | R-ICS | 2.92±0.08 | 2.68±0.64 | 2.76±0.38 | 2.54±0.78 |
|  | R-ACS | 2.19±0.32 | 1.95±0.04 | 2.03±0.66 | 2.33±0.64 |
| Lactic acid bacteria | R-C | <2 | <2 | <2 | <2 |
|  | R-ICS | <2 | <2 | <2 | <2 |
|  | R-ACS | <2 | <2 | <2 | <2 |
| *Enterobacteriaceae* | R-C | <2 | <2 | <2 | <2 |
|  | R-ICS | <2 | <2 | <2 | 2.63±1.22 |
|  | R-ACS | <2 | <2 | <2 | <2 |
| *L. monocytogenes* | R-C | n.d. | n.d. | n.d. | n.d. |
|  | R-ICS | n.d. | n.d. | n.d. | n.d. |
|  | R-ACS | n.d. | n.d. | n.d. | n.d. |

Note(s): R-C = Control restructured salmon (no antioxidants); R-ICS = Restructured salmon with industrial coffee silverskin; R-ACS = Restructured salmon with artisanal coffee silverskin. Results are expressed as mean ± standard deviation (n = 3). Values expressed as < 2 indicate that the microbial counts were below the quantification limit of the microbiological method (2 log CFU/g). n.d. = not detected in 25 g (qualitative analysis according to ISO 11290-1).

Supplementary Figure S1. Representative optical microscopy images showing the morphological structure of the milled coffee silverskin particles: (A) artisanal coffee silverskin (ACS) and (B) industrial coffee silverskin (ICS).
